# Six1-Eya1 Axis Governs Myofiber Remodeling and Fibrosis in Extraocular Myopathy: Insights from Single-Cell RNA Sequencing and Mesenchymal Stem Cell Therapy in Thyroid Eye Disease

**DOI:** 10.3390/cells14211708

**Published:** 2025-10-31

**Authors:** Hyun-Ah Shin, Mira Park, Hey Jin Lee, Jong Hyun Moon, Jasvinder Paul Banga, Helen Lew

**Affiliations:** 1 Department of Biomedical Science, CHA University, Seongnam-si 13488, Gyeonggi-do, Republic of Korea; sha9547@naver.com (H.-A.S.);; 2 Department of Ophthalmology, Bundang CHA Medical Center, CHA University, Seongnam-si 13496, Gyeonggi-do, Republic of Korea; hoohoo99@chamc.co.kr; 3 CHA Advanced Research Institute, CHA University, Seongnam-si 13488, Gyeonggi-do, Republic of Korea; 4 Molecular Ophthalmology, Department of Ophthalmology, University Duisburg-Essen, 45117 Duisburg, Germany

**Keywords:** thyroid eye disease (TED), orbital extraocular muscles, Six1, Eya1, myopathy, myofiber, myofibroblasts, hyaluronan

## Abstract

**Highlights:**

**What are the main findings?**

- The Six1/Eya1 complex regulates the composition of type IIA, IIX/IIB and IIB myofibers in thyroid eye disease.
- FGFR1 and TGFβ signaling modulate the differentiation between myofibroblasts and fibroblasts in thyroid eye disease.

**What are the implications of the main findings?**

- Targeting Six1/Eya1 signaling may provide a novel therapeutic strategy to control muscle hypertrophy and adipogenesis in thyroid eye disease.
- Human mesenchymal stem cell therapy has the potential to reverse pathological fibrosis and restore extracellular matrix composition in thyroid eye disease.

**Abstract:**

Thyroid eye disease (TED) is an autoinflammatory condition characterized by fibrosis in orbital fat and extraocular muscles, primarily driven by TSH receptor antibodies and inflammatory cytokines. While research has predominantly focused on the involvement of fat tissue, the understanding of myopathy in TED remains limited. This study developed a TED mouse model and isolated myoblasts from both control individuals and TED patients for analysis. Single-cell RNA sequencing was used to investigate myofiber type changes in TED and their alterations following treatment with human-derived mesenchymal stem cells. Key regulatory genes involved in myofiber differentiation and fibrosis in myofibroblasts were identified, and their expression balance was validated in myoblasts derived from both normal individuals and TED patients. Our analysis revealed a disease-associated shift in myofiber types and identified Six1 and Eya1 as central regulators of myofiber differentiation and fibrosis suppression. These regulatory effects were validated in primary myoblasts isolated from both control and TED patients. Collectively, our findings uncover a novel role for the Six1/Eya1 axis in modulating muscle remodeling and fibrosis in TED and provide a foundation for the development of targeted therapies for TED-associated myopathy.

## 1. Introduction

Thyroid eye disease (TED), also known as Graves’ ophthalmopathy, is characterized by chronic inflammation of the orbital extraocular muscles (EOMs) and fat tissues, typically in association with autoimmune thyroid disease [1,2]. TED is mediated by antibodies targeting the thyroid-stimulating hormone receptor (TSHR) and the insulin-like growth factor-1 receptor (IGF1R), which leads to hyperthyroidism. The disease is marked by enlargement of the EOM due to fat accumulation, inflammation-related hyaluronan (HA) deposition, and fibrosis. These pathological changes result in myopathy, which manifests as proptosis, eyelid retraction, ophthalmoplegia, and diplopia, causing significant functional impairments [3].

Thy-1 (CD90) is a critical marker of TED orbital fibroblasts (TED-OF), initially characterized in early studies and now widely recognized as the primary cellular effectors in TED [4]. These fibroblasts exhibit heterogeneity, comprising Thy1-positive (CD90+, Thy1+) and Thy1-negative (CD90−, Thy1−) subpopulations, which have distinct morphological characteristics and differentiation potentials [5]. The Thy1+ phenotype demonstrates the capacity to differentiate into myofibroblasts when activated through the transforming growth factor-beta (TGF-β) pathway. In contrast, Thy1− TED-OF are more inclined to differentiate into adipocytes via activation of the peroxisome proliferator-activated receptor gamma (PPARγ) pathway [6,7]. These findings highlight the role of Thy1+ OFs in promoting fibrosis in orbital tissue, a hallmark of late-stage TED morbidity. Thy1+ myofibroblasts contribute to myopathy, which manifests as diplopia or strabismus [8].

Myopathy, or muscle disease, affects muscle fiber function and leads to muscle weakness [9]. Most muscles are composed of multinucleated fibers that facilitate movement and include various myofiber types with distinct metabolic and functional properties. Type II fibers (MyHC-II or fast-twitch) are categorized into three subtypes: IIA, IIX, and IIB. In the context of ocular function, slow eye movements and gaze fixation are predominantly mediated by Type I myofibers, while rapid eye movements (saccades) and coordinated eyelid movements required for binocular fusion are facilitated by Type II myofibers. Muscle fibers exhibit considerable variability in their contractile and metabolic properties [10].

In TED pathophysiology, the interaction between IGF1R and TSHR has been shown to stimulate HA and glycosaminoglycan production, triggering inflammation and the differentiation of myofibroblasts, primarily driven by the TGF-β pathway [11,12,13]. This cascade leads to fibrosis and HA accumulation, ultimately impairing muscle function. Despite substantial research on TED, most studies have focused on fat tissue hypertrophy, with relatively limited attention given to myopathy, which remains insufficiently explored.

In a previous study, we demonstrated the reduction in fat volume in TED-OF and TED animal models using human mesenchymal stem cell (hMSC) therapy [14]. Human MSCs were selected for their established regenerative and immunomodulatory properties, including their ability to secrete trophic factors, attenuate inflammation, and influence fibrotic and adipogenic processes in diseased tissues [15]. By applying hMSC therapy in our TED model, we aimed to determine whether this intervention could reverse disease-associated alterations in myofiber type composition and suppress fibrotic remodeling of extraocular muscles. Our goal was to elucidate the molecular and cellular mechanisms underlying TED-associated myopathy and to evaluate the therapeutic potential of hMSCs in restoring normal muscle architecture. Following hMSC injection and single-cell RNA sequencing (scRNA-seq) analysis, we identified novel genes associated with myofiber specification and differentiation in TED myopathy. Consistent with our hypothesis, hMSC treatment partially restored the balance of key regulatory pathways, normalized the distribution of myofiber types, and attenuated fibrotic remodeling, highlighting the potential of hMSCs as a targeted therapeutic strategy for TED-associated extraocular myopathy.

## 2. Materials and Methods

### 2.1. Development of an Experimental Animal Model of TED Using Female BALB/c Mouse

Six-week-old female BALB/c mice (KOATEC, Seongnam-si, Gyeonggido, Republic of Korea) were used for experiments conducted in accordance with protocols approved by the Institutional Animal Care and Use Committee of Bundang CHA Medical Center (IACUC No. 230128). We generated a TED animal model by injecting pTriEx1.1Neo-hTSHR A-subunit plasmid into leg muscle using electroporation and characterized TED model in our previous report [1,16]. The animals with experimentally induced TED (thyroid eye disease) were injected with hMSCs (3 × 10^5^ cells/30 µL) under anesthesia with 3% isoflurane (Hana Pharm, Seoul, Republic of Korea). Animals were euthanized at one and two weeks after hMSC injection by carbon dioxide (CO_2_) inhalation, following ethical guidelines for animal experimentation. Euthanasia was performed in a sealed chamber with CO_2_ introduction at a flow rate displacing 30–70% of the cage volume per minute (mouse cage: 4–6 L/min).

### 2.2. Measurement of M22 Inhibition and T4 Levels

Validation of TED animal models was performed through serum analysis following a 9-week immunization period using the anti-TSHR antibody (TRAb) Fast ELISA kit (Euroimmun, Lübeck, Germany). The assay followed the manufacturer’s protocol, and results were expressed as the percentage inhibition of labeled M22 binding to immobilized TSHR in plate wells. Serum T4 levels were measured using the Total Thyroxine (T4) ELISA kit (DRG International, Springfield, NJ, USA), according to the manufacturer’s instructions.

### 2.3. TSHR Antibody Assay

The activities of TSAb and TSBAb were assessed through a bioassay using CHO cells stably transfected with human TSHR. CHO cells (30,000 cells/well) were seeded into 96-well flat-bottom plates. After 24 h, the medium was replaced with NaCl-free isotonic HBSS (Hanks’ balanced salt solution) containing sucrose, HEPES, and 0.5 mM isobutyl-1-methylxanthine (Sigma-Aldrich, St. Louis, MO, USA) to inhibit phosphodiesterase activity. Parallel assays were conducted in isotonic HBSS, with 130 mM NaCl replacing sucrose. Bovine TSH (bTSH; 40 μU/mL), test serum (3 μL), or purified IgG diluted in the appropriate HBSS was added in triplicate and incubated for 4 h at 37 °C. The cAMP released into the medium was measured using the Mouse/Rat cAMP kit (R&D Systems). Remaining procedures followed the reference protocol [17]. Animals exhibiting > 65% inhibition of labeled TSH binding activity compared to normal controls were included in the study. Experimental groups comprised TED-hMSCs-treated animals (3 × 10^5^ cells/30 µL) and untreated TED animals (BSS 30 µL). Intra-orbital injections were performed on the left orbit. Tissue analyses were conducted at 1 and 2 weeks following hMSC injection.

### 2.4. Orbital Tissue Histopathology and Immunofluorescence

Whole eyeballs were fixed with 4% paraformaldehyde, dehydrated in ethanol, and embedded in paraffin. Paraffin-embedded blocks were sectioned into 5-μm slices for histological analysis, including hematoxylin and eosin (H&E) and Masson’s trichrome (MT) staining. Orbital muscle area around the optic nerve was quantified using a ZEISS Axio Scan.Z1 slide scanner (Carl Zeiss, Jena, Germany), normalizing cross-sectional muscle area to the contralateral orbital tissue. Muscle areas from each group were evaluated. Primary antibodies for immunofluorescence included biotin-conjugated HABP (1:100, Amsbio, Abingdon, UK, Catalog No. 400763), anti-ITIH1 (1:200, Thermo Fisher Scientific, Waltham, MA, USA, Catalog No. PA1-1755), and anti-ITIH2 (1:200, Novus Biologicals, Centennial, CO, USA, Catalog No. NBP2-31750). Secondary antibodies were anti-Streptavidin (Vector Laboratories, Newark, CA, USA, Catalog No. BA-0500-5) and HRP donkey anti-rabbit IgG (Thermo Fisher Scientific).

### 2.5. scRNA-Seq and Data Processing

Orbital tissue was excised 1 week after hMSC or BSS injection for single-nuclei RNA sequencing. Libraries were prepared using the 10× Chromium Next GEM Single Cell 3′ v3.1 protocol (10× Genomics, Pleasanton, CA, USA). Raw sequencing data were assessed for quality using FastQC and processed using the Cell Ranger v7.0.1 count pipeline (10× Genomics) with the mouse genome (GRCm39). Downstream snRNA-seq data analysis was conducted using R v4.3.2 [18] and the Seurat package v4.3.0 [19].

### 2.6. Cell Type Annotation

Filtered data were normalized using the SCTransform package, which uses a negative binomial regression model with regularized parameters. PCA was performed using Seurat’s “RunPCA” function, and the top 30 principal components were used for clustering analysis via shared nearest neighbor modularity optimization. Uniform Manifold Approximation and Projection (UMAP) was used for cell cluster visualization. Marker identification in each cluster was conducted using the “FindAllMarkers” function, with cell type identities assigned based on canonical markers from recent literature.

### 2.7. Sub-Clustering Analysis and Single-Cell Gene Set Scoring

Myocytes and fibroblasts (including fibroblast and myofibroblast subpopulations) were isolated from the scRNA-seq data for sub-clustering. Data integration followed established protocols. The “AddModuleScore” function in Seurat was used to calculate single-cell expression levels, with gene sets derived from well-known cell type markers.

### 2.8. Functional Enrichment Analysis

Differentially expressed genes (DEGs) were identified using the “FindMarkers” function in the Seurat R package (logfc.threshold = 0.25). Functional enrichment analysis was conducted using the R package clusterProfiler v4.13.0 [20], incorporating Gene Ontology (GO) Biological Process [21] and Kyoto Encyclopedia of Genes and Genomes (KEGG) terms [22]. Statistical significance was defined as *p* < 0.05.

### 2.9. Constructing Single-Cell Trajectories

Single-cell trajectories were analyzed using the Monocle2 package (v2.30.1) [23] to identify cell-state transitions. Gene expression correlation with pseudotime in each trajectory was tested using Monocle. Dimensionality reduction was achieved with the “DDRTree” function, followed by cell ordering. The visualization of trajectories was performed using the “plot_cell_trajectory” function, while gene expression patterns were plotted using the “plot_genes_in_pseudotime” and “plot_genes_jitter” functions.

### 2.10. Cell–Cell Interaction Analysis

Cell–cell interactions were analyzed based on the expression of known ligand-receptor (L–R) pairs across cell types using CellChat (v2.1.2) [24] following the developer’s guidelines (https://github.com/sqjin/CellChat (accessed October 2024)). The snRNA-seq gene expression matrix served as input data, with “ChatDB.mouse” selected as the database. The “computeCommunProb” function was employed to infer the probability and strength of cell–cell communication. Visualization of communication networks was performed using circle, chord, and hierarchy plots, while signaling pathways were displayed as bubble plots.

### 2.11. Cell Isolation and Preparation of hMSCs and Human Myoblasts

The protocol for human myoblast isolation was approved by the Institutional Review Board (IRB 2018-01-007) of Bundang CHA Hospital, Seongnam, Republic of Korea, with informed consent obtained from all patients. EOM tissues were obtained during strabismus surgery from three control patients with alternating exotropia and three TED patients with restrictive strabismus (Supplementary Table S1). Myoblast isolation and culture methods were conducted as previously described [25,26]. Preparation of hMSCs followed the previously reported protocol [27]. The collection and use of hMSCs were approved by the Institutional Review Board of CHA General Hospital, Seoul, Republic of Korea.

### 2.12. Human MSCs Co-Culture Experiments

Before co-culture, myoblasts were pre-treated with 4-methylumbelliferone (4-MU; 400 nM, Thermo Fisher Scientific) for 30 min and IL1β (Peprotech, Rocky Hill, NJ, USA). hMSCs (2 × 10^5^ cells) were co-cultured with myoblasts using Transwell inserts (8 μm pore size; Corning, NY, USA) for 24 h at 37 °C in a humidified atmosphere containing 5% CO_2_.

### 2.13. Flow Cytometry

Cells were washed with 2% bovine serum albumin in phosphate-buffered saline and incubated with an antibody against CD90-PE (BioLegend, San Diego, CA, USA) on ice for 20 min. Fluorescence-activated cell sorting was performed using a CytoFLEX flow cytometer (Beckman Coulter, Brea, CA, USA) at a low flow rate with 10,000 events recorded. Data analysis was conducted using CytoExpert analysis software (Beckman Coulter, version accessed in 2024).

### 2.14. Quantitative Real-Time Polymerase Chain Reaction (qRT-PCR)

Total RNA was isolated from human myoblasts using TRIzol reagent (Ambion, Carlsbad, CA, USA), and cDNA synthesis was performed with 1 μg of RNA following the manufacturer’s instructions. Gene expression was quantified using the delta-delta CT method with amfiSure qGreen Q-PCR Master Mix, Low ROX (GenDEPOT, Katy, TX, USA). Real-time PCR reactions were conducted on a QuantStudioTM1 Real-Time PCR Instrument (Applied Biosystems, Foster City, CA, USA). The sequences of primers used are provided in Supplementary Tables S2 and S3.

### 2.15. Low-Molecular-Weight (MW) and Medium-High MW HA Detection Analysis

Culture media were harvested 24 h post IL1β exposure, and low MW and medium-high MW HA levels were measured using ELISA kits: Hyaluronan DuoSet ELISA Kit (R&D Systems, Minneapolis, MN, USA) for low MW HA and General Hyaluronic Acid ELISA Kit (Biorbyt, Cambridge, UK) for medium-high MW HA, following the manufacturer’s instructions. Absorbance at 450 nm was measured using a microplate reader (Molecular Devices, San Jose, CA, USA).

### 2.16. Small Interfering RNA (siRNA) for Eya1

Eya1 protein knockdown was achieved using siRNA (Bioneer, Yuseong-gu, Daejeon, Republic of Korea) with the target sequence 5′-UUG UGA GUG AAU UAU UUC CUG. A scrambled siRNA was used as a negative control. Transfections were performed in control and TED myoblasts using Lipofectamine 3000 (Thermo Fisher Scientific) according to the manufacturer’s protocol.

### 2.17. Six1 Overexpression and Knockdown

For Six1 overexpression, recombinant protein (Novus Biologicals) was used, while knockdown was achieved with an inhibitor drug (NCGC00378430, MCE, Monmouth Junction, NJ, USA). Normal and TED myoblasts were treated and incubated at 37 °C for 24 h.

### 2.18. Statistical Analysis

All results were presented as mean ± standard error of the mean (SEM). Statistical analyses were performed using GraphPad Prism 9 software (GraphPad Software, La Jolla, CA, USA). One-way analysis of variance (ANOVA) with Tukey’s post hoc test was used for normally distributed data, while the Mann–Whitney U test was applied to non-parametric data. Statistical significance was defined as *p* < 0.05. Details of statistical criteria are described in the figure legends.

## 3. Results

### 3.1. Characterization of the TED Animal Model

**Figure 1:**
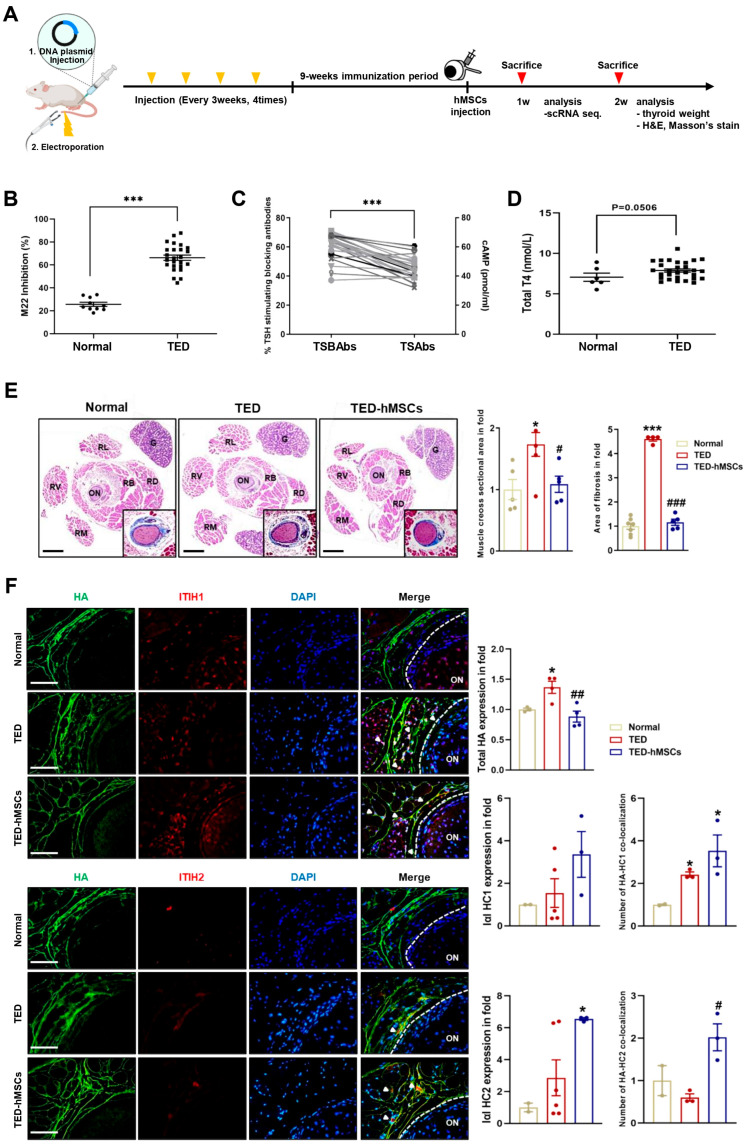
Characterization of the TED in animal model. (**A**) Experimental scheme for the in vivo construction of the TED animal model and the injection of hMSCs into TED animal (TED−hMSCs). (**B**) Anti-TSHR Ab inhibition (**C**) TSBAbs and TSAbs in serum of disease mice at 9 weeks after last immunization with pTriEx−1.1 TSH receptor (TSHR) A-subunit plasmid in muscle combined with electroporation. (**D**) Total T4 levels (**E**) Two weeks after the injection of hMSCs in TED model, an expansion of muscle cross−section area was observed, in addition to a representative histologic image of an orbital section, with H&E stained with Masson’s trichrome was used to comparing normal, TED and TED−hMSCs groups. The percentage of stained area in the muscle tissue, excluding the optic nerve and RB areas was measured. The main image is an H&E stained photograph, while the Masson’s trichrome stained image was magnified, cropped, and displayed in the bottom-right corner of the main image. Data was presented as a fold change (means ± SEM) of muscle area volume and area of fibrosis. Rectus Lateralis, RL; Retractor Bulbi, RB; Rectus Dorsalis, RD; Rectus Ventralis, RV; Rectus Medialis, RM; Optic Nerve, ON; Gland, G. Scale bar: 100μm. (**F**) Immunofluorescence staining of HA (red) and I−α−I HC1 and HC2 (green) in normal, TED and TED−hMSCs groups. The white arrowheads refer to the co-localization of HA and I−α−I HC1 and HC2, which means HA cross−linking. The intensity ratio of total HA and I−α−I HCs and counts of HA co−localized with I−α−I HCs to total HA were used to determine HA-HC complexes. Scale bar: 100μm. Significantly different values between the groups are indicated with markers (* *p* < 0.05, *** *p* < 0.001 vs. normal, # *p* < 0.05, ## *p* < 0.01, ### *p* < 0.001 vs. TED) normal *n* = 6, TED *n* = 12, TED−hMSCs *n* = 12.

We developed a TED animal model by electroporating human TSHR A-subunit plasmid (Figure 1A). After immunization, serum was analyzed for anti-TSHR antibodies. TED animals showed > 65% inhibition of labeled TSH binding in a TBII assay (Figure 1B), with positive anti-TSHR antibody subtypes (TSAbs: 17–58 pmol/mL; TSBAbs: 38–58% TSH-stimulating blocking ability) (Figure 1C). Clinical symptoms of orbital disease, such as redness, pus, and swelling, were observed in TED, improving after hMSC injection (TED-hMSCs) (Supplementary Figure S1A). Thyroid hormone T4 levels were similar in TED and normal groups, but thyroid weight and morphology were significantly increased in TED, with no change in TED-hMSCs (Figure 1D and Supplementary Figure S1B). Histology showed larger RB and RL areas in TED (173.4% larger than normal) and a 64.8% reduction in muscle area after hMSCs injection. Fibrosis increased by 452% in TED but decreased by 335.5% after hMSCs treatment (Figure 1E). Gene expression analysis showed elevated inflammation (Icam-1, Tnfα) and fibrosis markers (Tgfβ2, Vimentin, Fn1) in TED, with reduced expression after hMSCs treatment (Supplementary Figure S1C).

Importantly, early reports have shown that HA plays a role in regulating immune response and inflammation. In various tissues, HA forms covalent bonds with the heavy chains (HCs, HC1 and HC2) of inter-α-inhibitor (IαI), a reaction catalyzed by tumor necrosis factor-stimulated gene-6 (TSG-6), which is suggested to have a positive impact on inflammation resolution. In inflammatory environments, HA levels increase, leading to the abnormal accumulation of HA fragments. The anti-inflammatory effects of HA are mediated by IαI HCs, which, when complexed with HA, exhibit anti-inflammatory properties [28,29]. Immunofluorescence analysis showed increased total HA levels in TED, with a decrease in TED-hMSCs. Conversely, HA-HC1 and HA-HC2 complexes were elevated in TED-hMSCs (Figure 1F). These findings suggested that IαI-HC covalent bonding in hMSCs organizes complexes, promoting an anti-inflammatory environment, potentially offering a therapeutic strategy.

### 3.2. Single-Cell RNA Analysis Identified EOMs in the TED Animal Model

**Figure 2:**
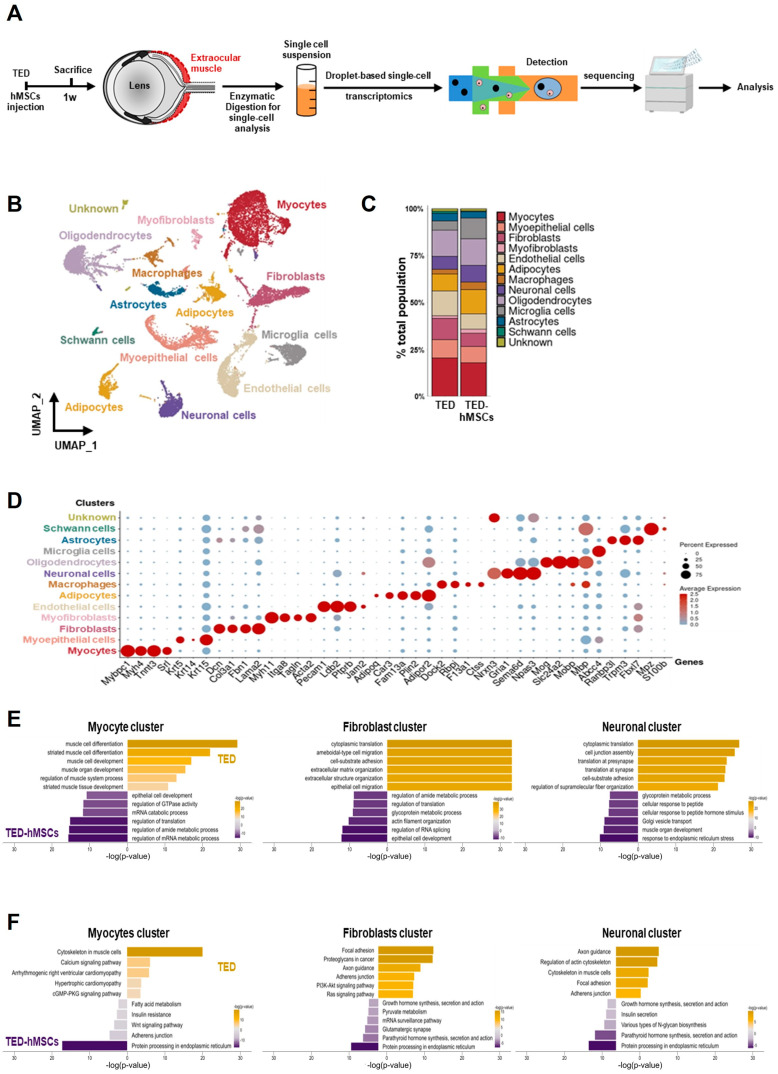
Single−cell RNA sequence analysis. (**A**) Overview of the experimental design. Cell suspensions were collected from the digested EOM surrounding the eyeball in TED and TED−hMSCs, followed by scRNA seq and downstream analyses. (**B**) A UMAP visualization illustrates the thirteen subpopulation cell type clusters. (**C**) Bar charts depict the proportions of subpopulation cell type clusters in TED and TED−hMSCs. (**D**) A dot plot represents the expression of selected cell type markers. As indicated by the legend, the diameter of each dot corresponds to the percentage of cells within a cluster expressing each gene (percent expression). (**E**) Biological Processes (BP) in Gene Ontology (GO) and (**F**) pathways in the Kyoto Encyclopedia of Genes and Genomes (KEGG) were analyzed for TED and TED−hMSCs in myocyte, fibroblast, and neuronal clusters.

To assess the cellular EOM composition in TED and the effects of hMSCs injection at 1 week, we performed scRNA-seq analysis. Single-cell suspensions from TED and TED-hMSCs were stained with a lipid-modified oligo hybridizing to DNA-barcoded samples and loaded into the 10X Genomics platform (Figure 2A). For quality control, cells with ≤ 200 genes, UMI counts < 1000, and >20% mitochondrial reads were excluded. After filtering, 15,474 cells from TED and 11,304 cells from TED-hMSCs were retained (Supplementary Figure S2A). UMAP visualization revealed thirteen distinct cell population clusters (Figure 2B), with cell proportion changes between TED and TED-hMSCs shown in a bar plot (Figure 2C). Cluster identities were confirmed by annotating based on the expression of known markers, visualized using dot plots (Figure 2D) and feature plots (Supplementary Figure S2B). We observed thirteen distinct cell clusters, including an unknown cluster, each identified by their unique signature genes: Mybpc1 (myocyte marker), Krt5 (myoepithelial cell marker), Col3a1 (fibroblast marker), Myh11 (myofibroblast marker), Pecam1 (endothelial marker), Plin2 (adipocyte marker), Dock2 (macrophage marker), Gria1 (neuronal cell marker), Mog (oligodendrocyte marker), Abcc4 (microglia marker), Trpm3 (astrocyte marker), and Mpz (Schwann cell marker). We also used GO and KEGG analyses to identify the predominant signaling pathways in myocyte, fibroblast, and neuronal clusters between TED and TED-hMSCs (Figure 2E,F).

In the myocyte cluster in TED, significantly enriched GO terms included muscle cell differentiation, development, and regulation of muscle system processes. In the TED-hMSCs myocyte cluster, enriched terms included regulation of mRNA metabolism, translation, and epithelial cell development. The fibroblast cluster in TED was enriched for cytoplasmic transition and cell–substrate organization, while TED-hMSCs fibroblast clusters showed similar enrichment, with additional terms related to epithelial cell development and translation regulation. In the neuronal cluster in TED, GO terms included cell junction and synaptic translation, whereas in TED-hMSCs neuronal clusters, enriched terms focused on ER stress response and metabolism. KEGG analysis revealed that TED myocyte clusters were associated with cytoskeletal regulation, while TED-hMSCs clusters were enriched for protein processing in the ER. TED fibroblast clusters focused on focal adhesion, similar to TED-hMSCs myocyte clusters. TED neuronal clusters highlighted axon guidance and actin cytoskeleton regulation, with TED-hMSCs pathways corresponding to those in myocyte, fibroblast, and neuronal clusters. These findings indicated the presence of diverse cell types in the TED-EOM, each associated with distinct functions and pathways.

### 3.3. Myocyte Sub-Clustering and Alterations in Myofiber Types Between TED and TED-hMSCs

**Figure 3:**
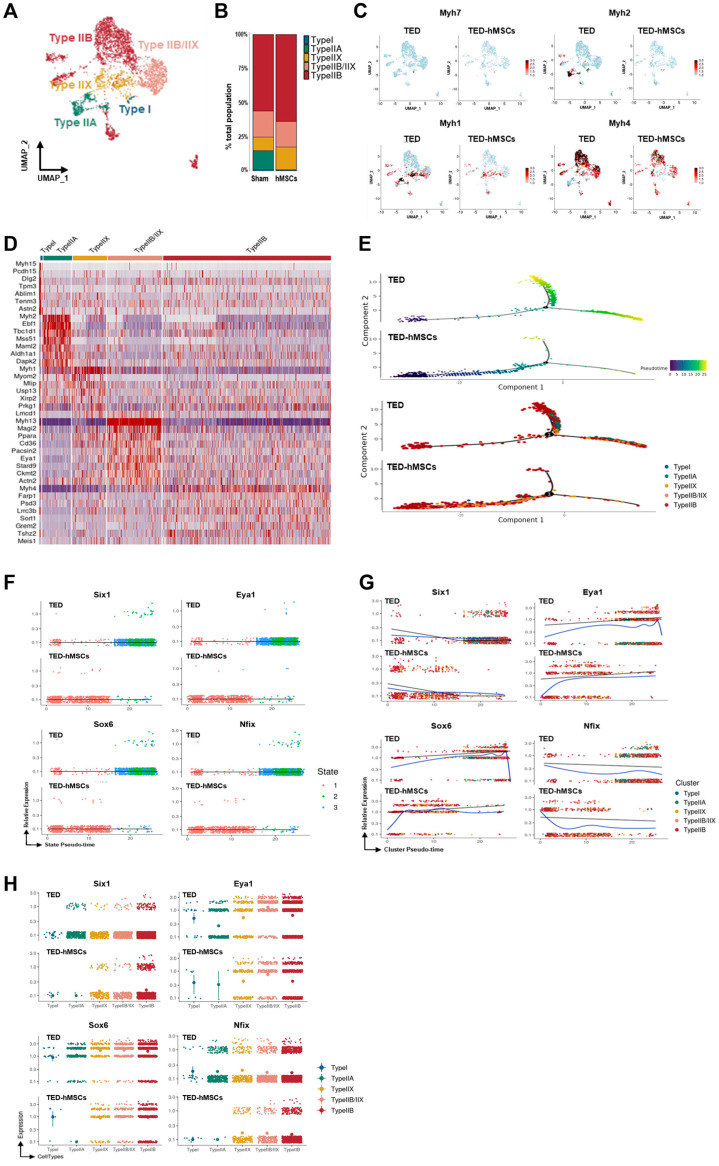
Single−cell RNA sequence analysis in the subcluster of myocytes. (**A**) A UMAP visualization displays subclusters of myocytes. (**B**) Bar charts illustrate the proportions of subpopulation cell types in TED and TED−hMSCs within the myocyte population. (**C**) A feature plot and (**D**) a heatmap show the differential expression of marker genes for myofiber types between TED and TED−hMSCs. The color scale represents z−score transformation of log2 values. (**E**) A Monocle2−based pseudotime trajectory illustrates the progression of myocyte differentiation between TED and TED−hMSCs. The color gradient represents the pseudotime score (upper panel), while different clusters are highlighted in the lower panel. Scatter plots depict changes in the expression levels of target genes over pseudotime, organized (**F**) by states, and (**G**) by clusters in TED and TED−hMSCs. A blue line represents the trend line for gene expression levels over pseudotime for each gene. (**H**) The expression patterns of target genes across different myocyte types are visualized using a color gradient through Monocle−2.

To explore further the differences in myocytes, sub-clustering analysis was conducted (Figure 3A), and TED marker gene expression was evaluated using feature plots (Supplementary Figure S3A). Four distinct myofiber types—Type I, Type IIA, Type IIX, and Type IIB—were identified using the “addModuleScore” function (Supplementary Figure S3B). A bar plot revealed changes in the proportions of these myofiber types between TED and TED-hMSCs (Figure 3B). In TED, Type IIB and Type IIX myofibers were predominant, while all myofiber type clusters showed decreased populations in TED-hMSCs, with the Type IIA cluster notably reduced.

The expression levels of myofiber-specific markers, Myh7 (Type I), Myh2 (Type IIA), Myh1 (Type IIX), and Myh4 (Type IIB), were compared using feature plots (Figure 3C) and analyzed using Monocle2 software (Supplementary Figure S3C). Expression levels of all markers were significantly reduced in TED-hMSCs. Heatmap analysis further confirmed the reduction in myofiber marker expression in TED-hMSCs compared to TED (Figure 3D). Pseudo-time trajectory analysis using Monocle2 was used to investigate muscle differentiation (Figure 3E). Both TED and TED-hMSCs exhibited a single-branch trajectory; however, TED displayed distinct differences in gene differentiation time (upper panel) and clustering (lower panel) along the pseudo-time. In TED, myofiber marker genes transitioned through differentiation states 1, 2, and 3, whereas TED-hMSCs remained confined to state 1 (Supplementary Figure S3C–E). TED showed increased clustering dynamics across Types I, IIA, IIX, and IIB along the pseudo-time, with significant gene expression at *X*-axis positions 0 and +10 (blue line). In contrast, TED-hMSCs exhibited no cluster commitment and showed reduced expression at corresponding positions. These findings highlight significant differences in muscle differentiation between TED and TED-hMSCs.

### 3.4. Key Target Genes Implicated in the Regulation of Myocyte Differentiation

Myogenesis predominantly occurs during in utero development when myogenic progenitor cells differentiate into mature myofibers. This process is orchestrated by signaling molecules and transcription factors, including Pax7 and the myogenic regulatory factor (MRF) family—Myf5, Myod, and Myogenin (Myog)—which regulate muscle differentiation and proliferation to establish the muscle phenotype [30,31,32]. Studies have identified Six1 as a key regulator of muscle development. Additionally, the forced expression of the Six1/Eya1 cofactor in Type I myofibers can induce a transition into Type II myofibers [33,34,35,36,37].

DEG analysis comparing TED and TED-hMSCs revealed that Pax7, MRFs, and Myostatin were not expressed in either group, while Six1 and Nfix were downregulated, and Eya1 and Sox6 were upregulated in TED (Table 1). Compared with TED-hMSCs, Six1 expression in TED was decreased by 0.75-, 0.54-, and 0.69-fold in Type IIX, IIX/IIB, and IIB myofibers, respectively. Nfix expression was also reduced in TED by 0.04-, 0.11-, 0.21-, and 0.38-fold in Type IIA, IIX, IIX/IIB, and IIB myofibers, respectively. In contrast, Sox6 expression in TED was increased by 3.30-, 1.89-, 2.46-, and 2.46-fold in Type IIA, IIX, IIX/IIB, and IIB myofibers, respectively. Similarly, Eya1 expression was upregulated in TED by 0.188-, 0.49-, 1.72-, and 0.94-fold in Type I, IIX, IIX/IIB, and IIB myofibers, respectively. Previous studies have indicated that Six1 regulates Type II myofiber differentiation, with Eya1 acting as a cofactor to facilitate the transition from Type I to Type II myofibers [36]. Conversely, Type I myofibers are determined by suppressing the transcription factors Sox6 and Nfix. As the scRNA-seq data were generated from pooled samples of three animals per group, statistical comparisons were not performed; values in Table 1 represent relative fold changes.

Previous studies have reported several transcription factors contributing to myofiber development, including Sox6, Nfix, Six1, and Eya1 [38]. Pseudo-time analysis of Six1, Eya1, Sox6, and Nfix expression revealed that, in TED, these genes transitioned through differentiation states 2 and 3, while TED-hMSCs remained confined to state 1 (Figure 3F). TED demonstrated clustering dynamics across Types I, IIA, IIX, and IIB along the pseudo-time, with significant downregulation of Six1 and Nfix, and upregulation of Eya1 and Sox6 at *X*-axis positions +10 and +20 (blue line). Conversely, in TED-hMSCs, Six1 and Nfix were upregulated, while Eya1 and Sox6 were downregulated at corresponding positions (Figure 3G). Gene distribution across clusters varied significantly (Figure 3H). Overall, these findings suggested that the interaction between Six1 and Eya1 plays a pivotal role in the transition from Type I to Type II myofibers, providing critical insights into myofiber differentiation dynamics in TED and the therapeutic effects of hMSCs treatment.

### 3.5. Fibroblast Sub-Clustering and Major Signaling Regulated in TED and TED-hMSCs

**Figure 4:**
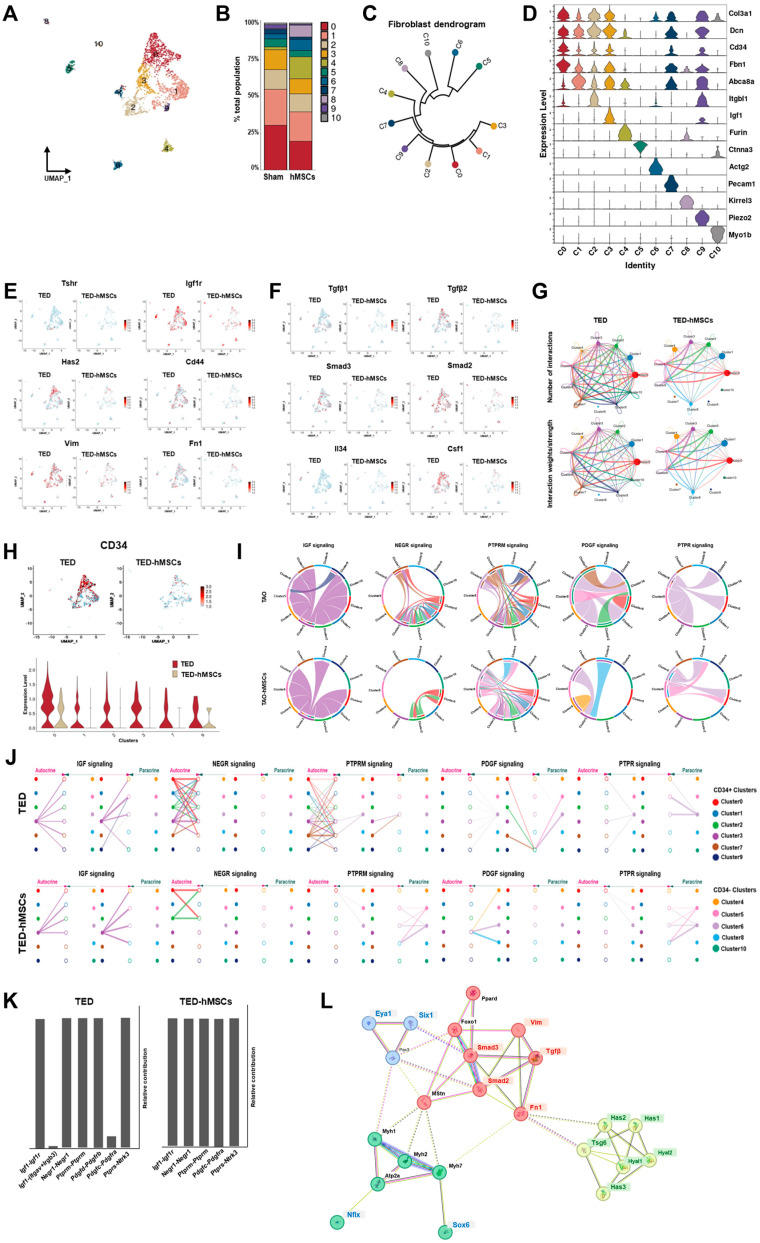
Single−cell RNA sequence analysis in the subcluster of myofibroblasts and fibroblasts. (**A**) UMAP representation visualized the eleven subpopulation cell type clusters. (**B**) Bar charts depict the proportion of subpopulation cell type clusters in TED and TED−hMSCs. (**C**) A dendrogram summarizes the similarities between cell subpopulations. (**D**) A violin plot illustrates the expression of selected markers for cell clusters. Feature plot depicts the expression of (**E**) TED-associated genes and (**F**) inflammation−related genes in TED and TED−hMSCs. (**G**) The number of interactions and strengths among all cell types were compared between TED and TED−hMSCs. (**H**) A feature plot (upper panel) and a violin plot (lower panel) show CD34−positive cell populations in TED and TED−hMSCs. (**I**) Chord diagrams represent cell−cell communication. The width of the edges corresponds to interaction strength, with thicker edges indicating stronger signals. Edge direction is shown from ligand to receptor, and the colors within the chord diagrams represent the corresponding cell or nuclei types. (**J**) A hierarchical plot illustrates the inferred intercellular communication network for five signaling pathways. This plot consists of two sections: the left portion highlights autocrine and paracrine signaling to CD34−positive fibroblast states, while the right portion focuses on CD34−negative fibroblast states. Solid circles represent sources, while open circles represent targets. The width of the edges represents the communication probability. (**K**) The relative contributions of each ligand-receptor pair mediating communication in the five signaling pathways. (**L**) STRING protein−protein interaction network.

To investigate the underlying mechanisms of TED, we performed sub-clustering of myofibroblasts and fibroblasts within the UMAP representation (Figure 2B). Using UMAP, we visualized the sub-clustering of fibroblasts (Figure 4A), and a bar plot diagram depicted the changes in the cell proportions of fibroblast clusters between TED and TED-hMSCs (Figure 4B). The dendrogram (Figure 4C) and heatmap (Figure 4D) illustrate the relationships and similarities among different subpopulations of cells. The dendrogram demonstrates the degree of similarity between cell subpopulations based on the analyzed data. Fibroblast characterization was further performed by examining TED-associated markers, myofibroblast markers (Figure 4E), and inflammation markers (Figure 4F) using feature plots for both TED and TED-hMSCs. Through subclustering of fibroblasts using scRNA-seq, we observed a significant upregulation of TGFβ and SMAD expression in TED, accompanied by increased expression of myofibroblast markers, such as vimentin and fibronectin. TED-hMSCs exhibited a decrease in the expression of these markers compared to TED across all genes.

### 3.6. CellChat Identified Communication Patterns and Predicted Functions for Understood Pathways

CellChat analysis was conducted to assess cell-to-cell communication across the whole cell population. The results of this analysis were visualized in interaction network plots, which showed the number of interactions and their respective weights (Figure 4G). Detailed networks for each cellular interaction were displayed in shell diagrams (Supplementary Figure S4A). CellChat also quantified the similarity between significant signaling pathways, grouping them based on their cellular communication network similarity, either by functional or structural similarity. Functional similarity grouping revealed four groups of pathways (Supplementary Figure S4B). In TED, Group #1 was dominated by cell adhesion pathways (e.g., PECAM1, SEMA3, and ADGRG). Group #2 consisted of growth factor pathways (e.g., VEGF, EGF, and IGF). Group #3 was associated with ECM component and fibrosis pathways (e.g., COLLAGEN, LAMININ, FN1, and PTPRM). Group #4 involved inflammatory and growth pathways (e.g., TGFβ, NOTCH, PDGF, and PTPR). In TED-hMSCs, Group #1 also included an ECM component and fibrosis pathways. Group #2 encompassed growth factor pathways. Group #3 was implicated in interactions between pre- and postsynaptic partners during synaptogenesis (e.g., NRG) and included cell growth, motility, and morphogenesis pathways (e.g., HGF). Group #4 again corresponded to the ECM component and fibrosis pathway. Compared to TED alone, TED-hMSCs exhibited markedly enhanced functional convergence across molecular pathways, with pathway clusters displaying significantly greater similarity in biological function.

### 3.7. CellChat Analysis of Communication Between CD34+ Clusters in TED and TED-hMSCs

Fibroblasts play a critical role in regulating the transition from acute resolving inflammation to chronic persistent inflammation, suggesting the potential contribution of CD34+ cells to the fibroblast pool within inflammatory microenvironments. Previous studies have reported that the CD34 + Thy1+ subset of fibroblasts exhibits high osteogenic and chondrogenic potential [39]. Moreover, in TED, the CD34+ fibroblast subset has been shown to induce the expression of inflammatory cytokines differently to that of the CD34− subset [40].

In our investigation, we examined the expression of CD34+ using feature plots (Figure 4H, upper) and violin plots (Figure 4H, lower) in both TED and TED-hMSCs. The analysis revealed that in TED, CD34+ expressions were higher in clusters 0, 1, 2, 3, 7, and 9, whereas TED-hMSCs showed a lower expression of CD34+ compared to TED. Furthermore, CellChat analysis was used to assess the ligand-receptor pairs in CD34+ clusters and other clusters, categorizing them into five signaling pathways (Figure 4H–J): IGF, NEGR, PTPRM, PDGF, and PTPR.

IGF emerged as a prominent signaling pathway in TED, while NEGR and PTPRM exhibited high signaling activity in the receiver (incoming signaling) clusters when the CD34+ clusters (0, 1, 2, 3, 7, 9) functioned as the sender (outgoing signaling). In contrast, PDGF and PTPR demonstrated elevated sender activity within the myofibroblast clusters (5, 6), distinguishing these signals from those associated with fibroblasts. Additionally, we identified the cell types mediating and influencing the five signaling pathways through network centrality analysis (Supplementary Figure S4C). The ligand-receptor pairs for each of the five signaling pathways were visualized in a heatmap (Supplementary Figure S4D).

Analysis of ligand-receptor (L-R) pairs revealed that, in TED, the L-R receptor pair Igf1-(Itgav + Irgb3) for the IGF signaling pathway and the L-R pair Pdgfd-Pdgfrb for the PDGF signaling pathway were absent in TED-hMSCs (Figure 4K). These findings indicated a general reduction in ligand-receptor expression across all groups in TED-hMSCs compared to TED.

### 3.8. Effect of hMSCs on Major Signaling Changes in ECMs in TED

Key ECM components, including COL1, LAMININ, and FN1, displayed significant cluster interactions in both TED and TED-hMSCs, as visualized in chord diagrams and hierarchical plots (Supplementary Figure S5A,B). However, these interactions were significantly diminished in TED-hMSCs. Network centrality analysis indicated reduced sender, receiver, mediator, and influencer signaling in TED-hMSCs compared to TED (Supplementary Figure S5C). L-R pair analysis of ECM-related signaling pathways further confirmed reduced signaling in TED-hMSCs (Supplementary Figure S5D,E). In TED, ECM components, such as VIMENTIN, COLLAGEN, and FN1, were actively expressed in fibroblasts, but showed significant reductions in TED-hMSCs. Additionally, inflammatory signaling in TED, we identified the inflammatory signaling pathway. FGF, EGF and TENASCIN were major components of inflammatory signaling pathway, acting in cluster interaction in TED, but decreasing cluster interaction in TED-hMSCs, as shown in chord diagrams (Supplementary Figure S6A), network centrality analysis (Supplementary Figure S6B) and hierarchical plots (Supplementary Figure S6C). Next, we examined the L-R pair of the inflammatory related-three signaling pathway using relative contribution (Supplementary Figure S6D) and expression of L-R pairs in each cluster using heat map (Supplementary Figure S6E). The analysis revealed that hMSCs reduced the L-R signals related to fibrosis, ECM components, and inflammatory signaling pathways in TED. These findings suggested that hMSCs effectively reduced fibrosis, ECM components, and inflammatory signaling, thereby supporting their potential as a therapeutic approach for TED. We used the STRING software v12.0 to analyze the protein–protein interaction network of SIX1 and EYA1 (Figure 4L). The analysis revealed interactions involving myofibers (e.g., Myh1, Myh2, Myh7, and Atp2a), fibrosis (TGFβ, Smad2, Smad3, Vim, and Fn1), and ECM components (Has1, Has2, Has3, Tsg6, Hyal1, and Hyal2) associated with proteins implicated in TED. Notably, SIX1 and EYA1 were found to form a central regulatory network with key TED-associated genes, highlighting their pivotal roles in the molecular pathogenesis of the disease.

### 3.9. Identification of Human Myoblasts Isolated from Control and TED Patients

**Figure 5:**
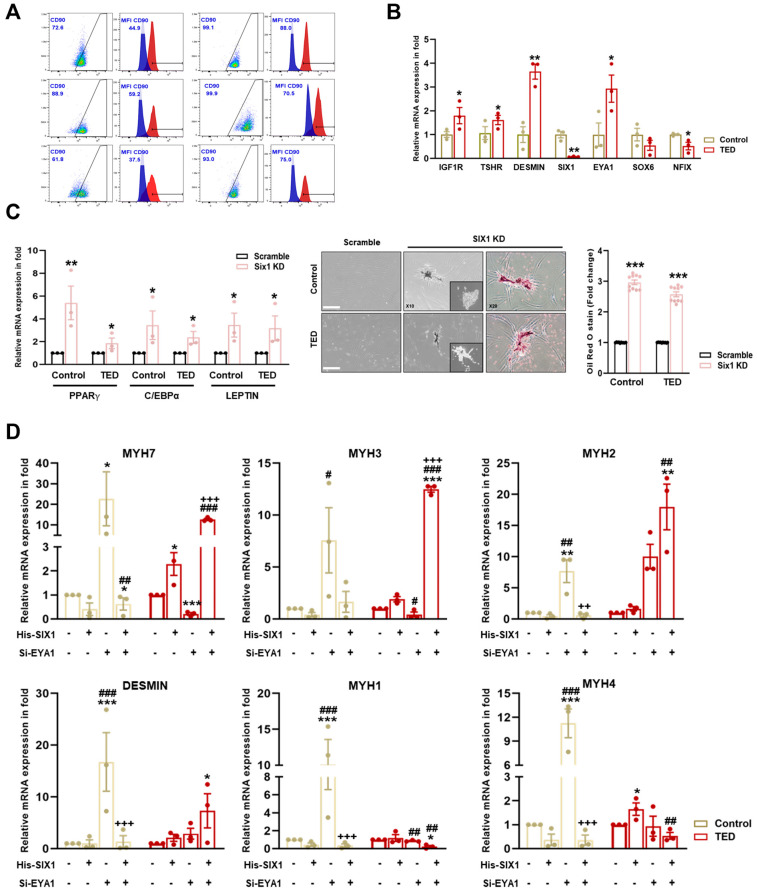
Characterization of myoblasts isolated from control and TED patients. (**A**) The expression of CD90 on the surface of control and TED myoblasts was measured by flow cytometry. (**B**) The relative mRNA expression levels of TED associated genes (e.g., IGF1R and TSHR), muscle marker genes (DESMIN), and myofiber differentiation genes (SIX1, EYA1, SOX6, and NFIX) were analyzed by qRT−PCR in myoblasts from control and TED samples (*n* = 3 for each group). (**C**) The SIX1 inhibition drug NCGC00378430 (10 μM) was treated for 24 h in control and TED myoblasts. Following treatment, the mRNA expression levels of adipogenesis-related genes (e.g., PPARγ, C/EBPα, and LEPTIN) were analyzed by qRT−PCR (left panel). Cell morphology was observed under a microscope after drug treatment, and Oil Red O (ORO) staining was performed (middle panel). Quantification of ORO staining in control and TED myoblasts was conducted, and the results were presented as relative absorbance at 470 nm (right panel). Scale bar: 200μm. (**D**) The expression of myofiber markers (e.g., MYH7, MYH3, MYH2, DESMIN, MYH1, and MYH4) was analyzed by qRT−PCR following individual or combined treatments of Six1 overexpression (5 μM) for 24 h and Eya knockdown (20 nM) for 72 h in both groups. Significantly different values between the groups are indicated with markers (* *p* < 0.05, ** *p* < 0.01, *** *p* < 0.001 vs. control, # *p*<0.05, ## *p* < 0.01, ### *p* < 0.001 vs. His-SIX1, ++ *p* < 0.01, +++ *p* < 0.001 vs. si-EYA1).

To characterize myoblasts from normal and TED patients (Supplementary Table S1), we first examined the heterogeneity of TED fibroblast cultures, which were identified as comprising both CD90+ and CD90− cells, exhibiting similar morphologies and growth characteristics. FACS analysis was performed to assess CD90 expression in myoblasts isolated from the EOMs of both control and TED patients. Increased expression of CD90+ was observed in TED patients compared to control individuals (Figure 5A). Based on these findings, TED myoblasts were classified as exhibiting characteristics indicative of the active subtype. We also examined the mRNA expression of thyroid-associated marker genes (e.g., IGF1R and TSHR), muscle markers (DESMIN), and myofiber differentiation-associated genes (e.g., SIX1, EYA1, SOX6, and NFIX). The results showed higher mRNA expression of IGF1R, TSHR, and DESMIN in TED patients compared to control myoblasts. Additionally, scRNA-seq results from TED showed decreased expression of SIX1 and NFIX, with increased expression of EYA1 (Figure 5B).

### 3.10. SIX1 Knockdown Induced Adipogenesis in TED

To assess further the impact of reduced SIX1 levels in TED, SIX1 knockdown was performed (Figure 5C). Knockdown of SIX1 resulted in the upregulation of adipogenesis-related genes, including PPARγ, C/EBPα, and LEPTIN. Additionally, ORO staining revealed increased lipid accumulation in TED following the loss of SIX1. These results highlighted SIX1 as a critical gene in the physiological regulation of TED and identified it as a key molecular target involved in the pathophysiology of the disease.

### 3.11. Effect of His-SIX1 and Knockdown of EYA1 on Myofiber Type

We further investigated gene expression alterations in myocytes through scRNA-seq analysis comparing control and TED. To explore the potential interaction between SIX1 and EYA1 in myofiber type switching, we performed SIX1 overexpression (which was downregulated in TED) and employed siRNA-mediated knockdown of EYA1 to assess the effects on myofiber markers (Figure 5D). Upon independent knockdown of EYA1, there was a significant increase in both Type I and Type II myofibers in control samples. In contrast, in TED, EYA1 knockdown resulted in a decrease in Type I and an increase in Type IIA myofibers, with no significant change in Type IIB myofibers. SIX1 overexpression alone did not significantly affect or lead to a decrease in slow-Type I and fast-Type II myofibers in controls. However, in TED, SIX1 overexpression showed a tendency to increase both slow-Type I and fast-Type II myofibers. When both EYA1 knockdown and SIX1 overexpression were combined, a significant reduction in Type IIX myofibers was observed in control samples. Interestingly, in TED, this combined treatment led to a significant increase in Type I and Type IIA myofibers, while Type IIX and Type IIB myofibers were significantly decreased. These findings suggested that the combined modulation of EYA1 and SIX1 facilitates myofiber type switching, contributing to the maintenance of myofiber balance in TED.

### 3.12. Effect of His-Six1 and Knockdown Eya1 on Ted Inflammation in Myofibroblasts

**Figure 6:**
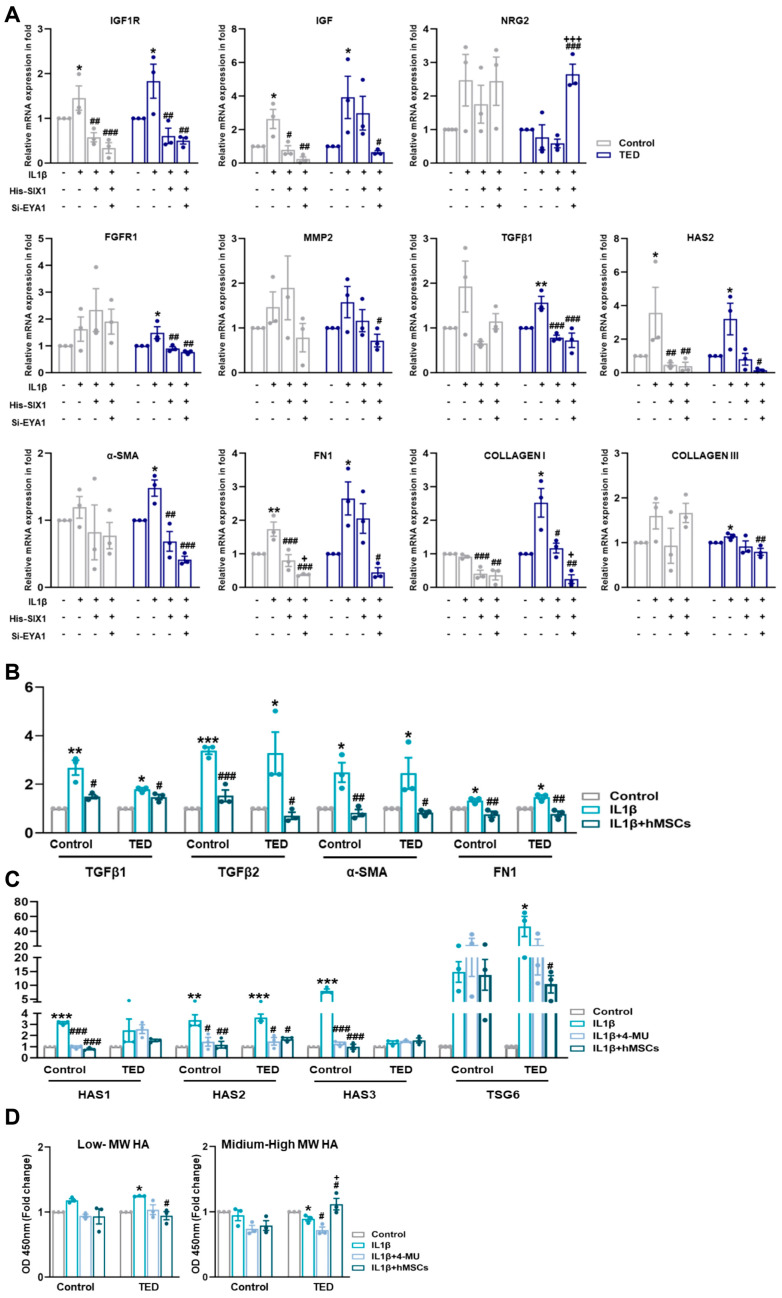
Nuanced regulations of SIX1 and EYA1 and its impact on myofibroblasts. (**A**) The expression of TED associated genes (e.g., IGF1R and IGF), neuronal genes (e.g., NRG2), fibrosis-related genes (e.g., FGFR1, MMP2, TGFβ2, and HAS2), and myofibroblast genes (e.g., α−SMA, FN1, COLLAGEN I, and COLLAGEN III) were analyzed by qRT−PCR 24 h after IL1β treatment (10 ng/mL) and following individual or combined treatments involving Six1 overexpression and Eya knockdown in control and TED myoblasts. (* *p* < 0.05, ** *p* < 0.01 control vs. IL1β, # *p* < 0.05, ## *p* < 0.01, ### *p* < 0.001 vs. His−SIX1, + *p* < 0.01, +++ *p* < 0.001 vs. si−EYA1). (**B**) The relative mRNA expression levels of inflammation−related genes (e.g., TGFβ1 and TGFβ2) and fibrosis−related genes (e.g., α−SMA and FN1 [FIBRONECTIN]) were analyzed by qRT−PCR after IL1β treatment and subsequent treatment with hMSCs. (**C**) The relative mRNA expression of hyaluronan (HA) synthase-related genes (e.g., HAS1, HAS2, HAS3) and TSG6 was analyzed by qRT−PCR following IL1β treatment and 30 min pre-treatments with 4−MU (400 nM/mL) and hMSCs. (**D**) Expression levels of HA molecules of varying sizes were measured in the culture media of control and TED using ELISA. Significantly different values between the groups are indicated with markers (* *p* < 0.05, ** *p* < 0.01, *** *p* < 0.001 control vs. IL1β, ## *p* < 0.01, ### *p* < 0.001 vs. IL1β, + *p* < 0.001 vs. IL1β + 4−MU).

To explore whether SIX1 and EYA1 modulate inflammation and myofibroblast marker genes, we induced an inflammatory state by treating cells with IL1β and then overexpressed SIX1 and performed EYA1 knockdown. Gene expression levels of relevant targets were subsequently assessed (Figure 6A). IL1β activation increased the expression of TED-associated genes IGF1R, TGFβ, and HAS2. However, modulation of SIX1 and EYA1 led to a reduction in these target genes. Additionally, IL1β-induced expression of myofibroblast markers, including α-SMA, FN1, COLLAGEN I, and COLLAGEN III, was diminished by SIX1 and EYA1 regulations. The upstream genes of TGFβ, FGFR1 and MMP2, were also upregulated by IL1β and downregulated upon modulation of SIX1 and EYA1. Functional analysis through CellChat further revealed that NGR2 in hMSC Group #3 was upregulated by SIX1 and EYA1. These data indicated that SIX1 regulates a large-scale differentiated cell identity program in TED by maintaining EYA1 binding at enhancers, which ultimately leads to a loss of myofiber pathogenesis. Moreover, these results suggested that SIX1 influences the expression of target genes involved in signaling pathways that mediate fibroblast inflammation and their subsequent differentiation into myofibroblasts. This highlighted SIX1 as a critical regulator of both cellular differentiation and pathological signaling in TED myopathy.

### 3.13. IL-1β Induced TED Inflammation and HA Synthesis-Related Gene Expression in Myoblasts

As shown in Figure 5A, TED represents an active subtype. We performed qRT-PCR to examine the effects of IL1β activation on inflammation, fibrosis, and HA synthesis. IL1β treatment of myocytes confirmed the upregulation of inflammation-related genes TGFβ1 and TGFβ2, with hMSCs specifically reducing TGFβ2 expression in TED. Fibrosis-related genes, α-SMA and FIBRONECTIN, increased in both control and TED myoblasts after IL-1β treatment, but were reduced only in hMSCs co-cultures. We also examined the effects of IL1β on HA synthesis-related gene expression. HAS2 expression was significantly higher in TED compared to controls (Figure 6B). To assess the effect of hMSCs, we compared changes in HAS synthesis-related genes following IL1β induction to the effects of 4-MU. Our results showed higher levels of HAS1, HAS2, and HAS3 expression in control cells, while HAS2 was most highly expressed in TED after IL1β treatment. Both 4-MU and hMSCs reduced the expression of HAS1, HAS2, and HAS3 in control cells, while only HAS2 expression was reduced in TED. These findings indicated that hMSCs specifically target HAS2, achieving a reduction equivalent to that of 4-MU. Furthermore, TSG-6 expression, which increased with IL1β exposure, was significantly reduced by hMSCs in TED, in contrast to 4-MU treatment (Figure 6C). ELISA of culture medium harvested after IL1β exposure showed increases in low and medium-high MW HA in both control and TED myocytes (Figure 6D). In TED, hMSCs reduced low-MW HA while increasing medium-high MW HA levels. These results suggested that hMSCs inhibit HA synthesis, inflammation, and fibrosis in TED more effectively than 4-MU. IL1β upregulated HAS2 in TED myoblasts, while 4-MU downregulated it. Human MSCs reduced HAS2 in TED and enhanced HA-IαI HC binding, while significantly downregulating the elevated TSG6 expression in TED myoblasts.

## 4. Discussion

TED is characterized by TSHR-expressing fibrocytes, which, upon activation by their ligand, produce elevated levels of inflammatory cytokines. Additionally, the IGF1R, expressed by OFs and fibrocytes, appears to be essential for TSHR-dependent cytokine production. TED is a progressive autoimmune disorder that leads to the enlargement of orbital fat and EOMs [41]. Teprotumumab, an IGF1R inhibitory monoclonal antibody, is currently the only FDA-approved drug for reducing the activity and severity of TED [42]. Teprotumumab inhibits the interaction with TSHR signaling by blocking the activation of IGF1R. In a clinical trial, 88 patients were randomly assigned to receive either a placebo or active drugs. The primary outcome was the response in the study eye compared with placebo. The trial demonstrated that 29 of the 42 patients (69%) who received teprotumumab had a response at week 24, while only 9 of the 45 patients (20%) who received placebo responded (*p* < 0.001) [43]. A significant reduction of 80% in proptosis and a 47% improvement in diplopia were observed in the treatment group [44]. Despite the efficacy of teprotumumab, surgical intervention remains the most effective treatment for addressing limited eyelid movement in TED.

Limited eye movement can result from excessive shortening of an EOM, which may occur due to excessive resection or tightening of the muscle. Excessive shortening can lead to movement restrictions, particularly in the opposite direction of the shortened muscle [45], a condition known as myopathy. Myofiber types are classified as Type I and Type II based on differences in myosin heavy chain gene expression. Fast fibers are further subdivided into Types IIA, IIX, and IIB based on metabolic properties [10,46]. Thyroid hormones have a significant impact on myofiber phenotypes, with hypothyroidism inducing a shift from Type II to Type I fibers, while hyperthyroidism causes the reverse shift. Additionally, reduced thyroid hormone levels trigger a transition from Type II to Type I in MHC (major histocompatibility complex) isoform expression [47]. Type I and Type IIA fibers are classified as oxidative due to their high metabolic capacity, while Type IIX and IIB fibers are classified as glycolytic [48].

The Six1 gene, a homolog of the Drosophila sine oculis gene, belongs to the Six gene family, which includes six members classified into three subgroups: Six1/Six2, Six3/Six6, and Six4/Six5. The Six gene family plays a critical role in organogenesis [49] and disease pathogenesis [50]. Among these, Six1 is essential for both embryonic myogenesis and adult muscle regeneration. The loss of Six1 promotes cell cycle arrest and differentiation [35] and leads to fetal death due to severe rib malformations and extensive muscle hypoplasia [34]. In our study, inhibition of Six1 was found to induce adipogenesis, thereby aggravating the progression of TED. This finding highlights the critical role of Six1 in regulating fiber-type transitions and further suggests that its interaction with Eya1 constitutes a key complex in modulating myofiber composition [51].

The Eya (eye absent) protein, initially identified in Drosophila, interacts with Six1. In mammals, four Eya genes (Eya1–4) exist, with the Six1/Eya2 complex playing a role in early myogenesis. Recent studies, however, have highlighted the Six1/Eya1 complex in the metabolic and contractile specialization of mature muscle fibers. Six1/Eya1 expression in the pre-placodal ectoderm promotes the formation of cranial sensory organs and ganglia, emphasizing their importance in organ differentiation and development [52]. Our findings suggest that the decreased expression of Six1 and the increased expression of Eya1 in TED myofibers may result in the formation of an abnormal Six1/Eya1 complex, which could contribute to the pathogenesis of TED by promoting maladaptive myofiber-type transitions and excessive fibroblast-to-myofibroblast differentiation. Given that the Six1/Eya1 complex is tightly regulated within muscle fibers, we also provide preliminary evidence that its differential enrichment in the myonuclei of fast and slow fibers may involve post-transcriptional regulatory mechanisms [37,53]. Aberrant expression of Six1/Eya1 is likely to influence muscle fiber-type specification, contractile function, and hypertrophic responses. Furthermore, overexpression of Six1 has been reported to activate TGF-β signaling in breast cancer, implying a potential profibrotic role of Six1. These findings prompted us to investigate the potential involvement of Six1 in NAFLD progression [54]. Collectively, these results indicate that abnormal regulation of Six1 may directly contribute to fibrotic remodeling and that modulation of Six1/Eya1 complex expression could represent a potential therapeutic approach to restore normal myofiber composition and attenuate fibrosis in TED.

HA, a key ECM component, plays a pivotal role in regulating inflammation and mediating various cellular and biochemical processes, with its effects dependent on its molecular mass. High MW HA (>500 kDa) promotes tissue integrity and suppresses inflammation, while low MW HA (<500 kDa) triggers a pro-inflammatory response [53]. In this study, we further analyzed the key signaling pathways involving CD34+ cells in TED, which have not been previously described. Five signaling pathways that differentiate fibroblasts were identified, with the insulin-like growth factor (IGF) signaling pathway being prominently represented. Additionally, signaling of Neuronal Growth Regulator (NEGR), a cell adhesion molecule involved in neuronal synaptic function and connectivity, drew significant attention. Previous studies have reported the activation of NEGR signaling in CD34+Thy1+ fibroblasts [55,56], suggesting that neuronal activity signaling may also contribute to the pathogenesis of TED. Furthermore, signaling of Protein Tyrosine Phosphatase Receptor (PTPRM), a member of the immunoglobulin family of adhesion molecules, was identified as another crucial signaling pathway. PTPRM, expressed in neurons, glia, and epithelial cells, appeared to play a role in the adhesion functions of various cells present in the EOMs in TED. Additionally, Platelet-Derived Growth Factor signaling was implicated as a key regulator in the differentiation of fibroblasts to myofibroblasts, highlighting the complex interaction between ECM components and inflammation signaling in TED fibroblasts.

Myofibroblasts exhibit ultrastructural features intermediate between fibroblasts and smooth muscle cells and are characterized by their ability to express contractile proteins, particularly α-smooth muscle actin (α-SMA) and fibronectin. Transforming Growth Factor Beta 1 (TGFβ1) has been shown to induce myofibroblast differentiation both in vitro and in vivo, although the regulatory mechanisms behind this process in TED remain unclear [57,58]. In our study, we investigated whether Six1 and Eya1, key regulators of myogenesis, modulate the progression of fibroblasts to the myofibroblast phenotype, a critical factor in TED myopathy. Our findings indicated that Six1 and Eya1 regulate fibroblast-to-myofibroblast differentiation by modulating fibroblast growth factor and matrix metalloproteinase-2, leading to reduced expression of downstream targets, including TGFβ and myofibroblast markers, such as α-SMA and fibronectin. These results suggested that regulating Six1 and Eya1 expression could serve as a therapeutic strategy for TED myopathy by preventing excessive fibroblast differentiation.

This study had several limitations that should be addressed in future research. First, there was no parallel investigation of both muscle cells and adipocytes, which could provide further insights into the genetic consistency among patients and the underlying pathophysiological processes. Second, clinical therapeutic antibodies were not included in the comparative analysis due to cost constraints. Instead, the study focused on evaluating the efficacy of stem cell-based therapies, which may offer a more cost-effective alternative. Third, gene expression and myofiber-specific protein markers were not analyzed in blood samples from a larger clinical cohort, an essential step in linking genetic profiles to disease manifestations. Finally, future studies could benefit from co-culturing muscle cells with immune cells from blood using microchip technology or organoid models. This approach would enhance our understanding of cellular interactions and disease progression in TED.

Despite these limitations, our findings highlighted the potential of hMSCs as a therapeutic agent for TED myopathy. The study also demonstrated the critical role of balanced Six1/Eya1 expression in TED’s molecular mechanisms. These insights could support the development of targeted therapies aimed at modulating Six1/Eya1 signaling to mitigate the pathological effects of TED.

## 5. Conclusions

Taken together, our findings provide new insights into the molecular mechanisms driving extraocular muscle remodeling in TED. We demonstrated that dysregulated Six1 and Eya1 expression promotes both maladaptive myofiber-type transitions and fibroblast-to-myofibroblast differentiation, contributing to fibrotic remodeling and impaired muscle function. Importantly, hMSCs therapy effectively attenuated fibrosis, restored myofiber composition, and partially restored muscle homeostasis, highlighting its therapeutic potential for TED-associated myopathy. These results suggest that modulation of the Six1/Eya1 axis may represent a promising therapeutic strategy for preventing or reversing myopathic changes in TED. Future studies should validate these mechanisms in clinical settings and explore the translational potential of hMSCs-based interventions for TED-associated muscle dysfunction.

## Figures and Tables

**Table 1:** Cluster-specific expression levels of muscle differentiation genes in the myocyte sub-cluster.

	**TED**				
	**Type I**	**Type IIA**	**Type IIX**	**Type IIX/IIB**	**Type IIB**
**Pax7**	0.3913	0.0053	0.0226	0.0511	0.0241
**Myf5**	-	-	0.0037	0.0019	0.0040
**Myod1**	-	0.0134	0.0075	0.0118	0.0107
**Myog**	0.0434	-	0.0075	0.0078	0.0093
**Mstn**	-	0.088	0.1584	0.1082	0.1769
**Six1**	-	0.1048	0.0566	0.0590	0.0918
**Eya1**	1.6086	0.6935	1.9622	4.3799	2.5375
**Sox6**	3.5217	3.2956	5.1132	5.7933	6.0569
**Nfix**	0.8260	0.6048	0.5169	0.4645	0.4691
	**TED-hMSCs**				
	**Type I**	**Type IIA**	**Type IIX**	**Type IIX/IIB**	**Type IIB**
**Pax7**	0.2857	-	-	0.0147	0.0158
**Myf5**	-	-	-	0.0049	0.0057
**Myod1**	-	-	-	-	-
**Myog**	-	-	-	-	0.0115
**Mstn**	-	-	0.2637	0.0539	0.1959
**Six1**	-	-	0.2252	0.1274	0.2997
**Eya1**	1.4285	1	1.4725	2.6568	1.5994
**Sox6**	5.7142	-	3.2197	3.3382	3.5965
**Nfix**	0.8571	-	0.5824	0.5882	0.7507

## Data Availability

The data on differential expression genes (DEGs) are included in this Supplementary Information. We used publicly available software/R packages in all the analyses. These are listed with appropriate citations in the methods.

## Supplementary Materials

The following supporting information can be downloaded at: https://www.mdpi.com/article/10.3390/cells14211708/s1. Figure S1. Features of TED in animal model. Figure S2. Single-cell RNA sequence analysis. Figure S3. Single-cell RNA sequencing analysis in myocytes. Figure S4. Single-cell RNA sequencing analysis in fibroblast and myofibroblast. Figure S5. ECM component signaling pathway in fibroblast and myofibroblast. Figure S6. Inflammatory signaling pathway in fibroblast and myofibroblast. Table S1. Basic characteristics of patients. Table S2. Human primer sequences using qRT-PCR. Table S3. Mouse primer sequences using qRT-PCR.

## References

[1] Lee A.C.H., Kahaly G.J. (2023). Pathophysiology of thyroid-associated orbitopathy. Best Pract. Res. Clin. Endocrinol. Metab..

[2] Ueland H.O., Neset M.T., Methlie P., Ueland G.A., Pakdel F., Rodahl E. (2023). Molecular Biomarkers in Thyroid Eye Disease: A Literature Review. Ophthalmic Plast. Reconstr. Surg..

[3] Schluter A., Flogel U., Diaz-Cano S., Gortz G.E., Stahr K., Oeverhaus M., Plohn S., Mattheis S., Moeller L.C., Lang S. (2018). Graves’ orbitopathy occurs sex-independently in an autoimmune hyperthyroid mouse model. Sci. Rep..

[4] Chiu H.I., Wu S.B., Tsai C.C. (2024). The Role of Fibrogenesis and Extracellular Matrix Proteins in the Pathogenesis of Graves’ Ophthalmopathy. Int. J. Mol. Sci..

[5] Smith T.J. (2023). Fibrocyte Participation in Thyroid-Associated Ophthalmopathy Suggests New Approaches to Therapy. Ophthalmic Plast. Reconstr. Surg..

[6] Koumas L., Smith T.J., Phipps R.P. (2002). Fibroblast subsets in the human orbit: Thy-1+ and Thy-1- subpopulations exhibit distinct phenotypes. Eur. J. Immunol..

[7] Sorisky A., Pardasani D., Gagnon A., Smith T.J. (1996). Evidence of adipocyte differentiation in human orbital fibroblasts in primary culture. J. Clin. Endocrinol. Metab..

[8] Chang E.L., Rubin P.A. (2002). Upper and lower eyelid retraction. Int. Ophthalmol. Clin..

[9] Simon A., Diedhiou N., Reiss D., Goret M., Grandgirard E., Laporte J. (2024). Potential compensatory mechanisms preserving cardiac function in myotubular myopathy. Cell. Mol. Life Sci..

[10] Talbot J., Maves L. (2016). Skeletal muscle fiber type: Using insights from muscle developmental biology to dissect targets for susceptibility and resistance to muscle disease. Wiley Interdiscip. Rev. Dev. Biol..

[11] Douglas R.S., Afifiyan N.F., Hwang C.J., Chong K., Haider U., Richards P., Gianoukakis A.G., Smith T.J. (2010). Increased generation of fibrocytes in thyroid-associated ophthalmopathy. J. Clin. Endocrinol. Metab..

[12] Lehmann G.M., Feldon S.E., Smith T.J., Phipps R.P. (2008). Immune mechanisms in thyroid eye disease. Thyroid.

[13] Li Z., Wang M., Tan J., Zhu L., Zeng P., Chen X., Xie L., Duan R., Chen B., Tao T. (2022). Single-cell RNA sequencing depicts the local cell landscape in thyroid-associated ophthalmopathy. Cell Rep. Med..

[14] Shin H.A., Park M., Banga J.P., Lew H. (2022). TGFbeta-Treated Placenta-Derived Mesenchymal Stem Cells Selectively Promote Anti-Adipogenesis in Thyroid-Associated Ophthalmopathy. Int. J. Mol. Sci..

[15] Shan S.L., Xiang F., Xin W., Ji S.L., Miribangvl A., Tian S., Yuan Y.W., Hong W.C. (2024). How mesenchymal stem cells transform into adipocyte: Overview of the current understanding of adipogenic differentiation. World J. Stem Cells.

[16] Park M., Banga J.P., Kim G.J., Kim M., Lew H. (2019). Human placenta-derived mesenchymal stem cells ameliorate orbital adipogenesis in female mice models of Graves’ ophthalmopathy. Stem Cell Res. Ther..

[17] Gilbert J.A., Gianoukakis A.G., Salehi S., Moorhead J., Rao P.V., Khan M.Z., McGregor A.M., Smith T.J., Banga J.P. (2006). Monoclonal pathogenic antibodies to the thyroid-stimulating hormone receptor in Graves’ disease with potent thyroid-stimulating activity but differential blocking activity activate multiple signaling pathways. J. Immunol..

[18] Shen B., Liu J., Wu D., Guo J. (2024). Evaluation of the safety and efficacy of high-dose rate brachytherapy for radiorecurrent prostate cancer: A systematic review and meta-analysis. Strahlenther. Onkol..

[19] Hao Y., Hao S., Andersen-Nissen E., Mauck W.M., Zheng S., Butler A., Lee M.J., Wilk A.J., Darby C., Zager M. (2021). Integrated analysis of multimodal single-cell data. Cell.

[20] Wu T., Hu E., Xu S., Chen M., Guo P., Dai Z., Feng T., Zhou L., Tang W., Zhan L. (2021). clusterProfiler 4.0: A universal enrichment tool for interpreting omics data. Innovation.

[21] Ashburner M., Ball C.A., Blake J.A., Botstein D., Butler H., Cherry J.M., Davis A.P., Dolinski K., Dwight S.S., Eppig J.T. (2000). Gene ontology: Tool for the unification of biology. The Gene Ontology Consortium. Nat. Genet..

[22] Ogata H., Goto S., Sato K., Fujibuchi W., Bono H., Kanehisa M. (1999). KEGG: Kyoto Encyclopedia of Genes and Genomes. Nucleic Acids Res..

[23] Trapnell C., Cacchiarelli D., Grimsby J., Pokharel P., Li S., Morse M., Lennon N.J., Livak K.J., Mikkelsen T.S., Rinn J.L. (2014). The dynamics and regulators of cell fate decisions are revealed by pseudotemporal ordering of single cells. Nat. Biotechnol..

[24] Jin S., Guerrero-Juarez C.F., Zhang L., Chang I., Ramos R., Kuan C.H., Myung P., Plikus M.V., Nie Q. (2021). Inference and analysis of cell-cell communication using CellChat. Nat. Commun..

[25] Park M., Nepali S., Lew H. (2020). Isolation and Characterization of Extraocular Muscle-Derived Muscle Progenitor Cells from Normal and Graves’ Orbitopathy Patients. Stem. Cells Dev..

[26] Park M., Kim H.C., Kim O., Lew H. (2018). Human placenta mesenchymal stem cells promote axon survival following optic nerve compression through activation of NF-kappaB pathway. J. Tissue Eng. Regen. Med..

[27] Lee H.J., Cha K.E., Hwang S.G., Kim J.K., Kim G.J. (2011). In vitro screening system for hepatotoxicity: Comparison of bone-marrow-derived mesenchymal stem cells and Placenta-derived stem cells. J. Cell. Biochem..

[28] Zhang G., He Y., Liu Y., Du Y., Yang C., Gao F. (2021). Reduced hyaluronan cross-linking induces breast cancer malignancy in a CAF-dependent manner. Cell Death Dis..

[29] Lauer M.E., Glant T.T., Mikecz K., DeAngelis P.L., Haller F.M., Husni M.E., Hascall V.C., Calabro A. (2013). Irreversible heavy chain transfer to hyaluronan oligosaccharides by tumor necrosis factor-stimulated gene-6. J. Biol. Chem..

[30] Cai C., Yue Y., Yue B. (2023). Single-cell RNA sequencing in skeletal muscle developmental biology. Biomed. Pharmacother..

[31] Hernandez-Hernandez J.M., Garcia-Gonzalez E.G., Brun C.E., Rudnicki M.A. (2017). The myogenic regulatory factors, determinants of muscle development, cell identity and regeneration. Semin. Cell Dev. Biol..

[32] Yu D., Cai Z., Li D., Zhang Y., He M., Yang Y., Liu D., Xie W., Li Y., Xiao W. (2021). Myogenic Differentiation of Stem Cells for Skeletal Muscle Regeneration. Stem Cells Int..

[33] Laclef C., Hamard G., Demignon J., Souil E., Houbron C., Maire P. (2003). Altered myogenesis in Six1-deficient mice. Development.

[34] Grifone R., Demignon J., Houbron C., Souil E., Niro C., Seller M.J., Hamard G., Maire P. (2005). Six1 and Six4 homeoproteins are required for Pax3 and Mrf expression during myogenesis in the mouse embryo. Development.

[35] Hsu J.Y., Danis E.P., Nance S., O’Brien J.H., Gustafson A.L., Wessells V.M., Goodspeed A.E., Talbot J.C., Amacher S.L., Jedlicka P. (2022). SIX1 reprograms myogenic transcription factors to maintain the rhabdomyosarcoma undifferentiated state. Cell Rep..

[36] Maire P., Dos Santos M., Madani R., Sakakibara I., Viaut C., Wurmser M. (2020). Myogenesis control by SIX transcriptional complexes. Semin. Cell Dev. Biol..

[37] Sakakibara I., Wurmser M., Dos Santos M., Santolini M., Ducommun S., Davaze R., Guernec A., Sakamoto K., Maire P. (2016). Six1 homeoprotein drives myofiber type IIA specialization in soleus muscle. Skelet. Muscle.

[38] Taglietti V., Maroli G., Cermenati S., Monteverde S., Ferrante A., Rossi G., Cossu G., Beltrame M., Messina G. (2016). Nfix Induces a Switch in Sox6 Transcriptional Activity to Regulate MyHC-I Expression in Fetal Muscle. Cell Rep..

[39] Noda S., Hosoya T., Komiya Y., Tagawa Y., Endo K., Komori K., Koga H., Takahara Y., Sugimoto K., Sekiya I. (2022). CD34(+)THY1(+) synovial fibroblast subset in arthritic joints has high osteoblastic and chondrogenic potentials in vitro. Arthritis Res. Ther..

[40] Lu Y., Atkins S.J., Fernando R., Trierweiler A., Mester T., Grisolia A.B.D., Mou P., Novaes P., Smith T.J. (2018). CD34- Orbital Fibroblasts from Patients with Thyroid-Associated Ophthalmopathy Modulate TNF-alpha Expression in CD34+ Fibroblasts and Fibrocytes. Investig. Ophthalmol. Vis. Sci..

[41] Smith T.J. (2020). Thyroid-associated ophthalmopathy: Emergence of teprotumumab as a promising medical therapy. Best Pract. Res. Clin. Endocrinol. Metab..

[42] Nie T., Lamb Y.N. (2022). Correction: Teprotumumab: A Review in Thyroid Eye Disease. Drugs.

[43] Smith T.J., Kahaly G.J., Ezra D.G., Fleming J.C., Dailey R.A., Tang R.A., Harris G.J., Antonelli A., Salvi M., Goldberg R.A. (2017). Teprotumumab for Thyroid-Associated Ophthalmopathy. N. Engl. J. Med..

[44] Ugradar S., Kang J., Kossler A.L., Zimmerman E., Braun J., Harrison A.R., Bose S., Cockerham K., Douglas R.S. (2022). Teprotumumab for the treatment of chronic thyroid eye disease. Eye.

[45] Ozkan S.B. (2016). Restrictive problems related to strabismus surgery. Taiwan J. Ophthalmol..

[46] Schiaffino S., Reggiani C. (2011). Fiber types in mammalian skeletal muscles. Physiol. Rev..

[47] Pette D., Staron R.S. (2000). Myosin isoforms, muscle fiber types, and transitions. Microsc. Res. Tech..

[48] Furuichi Y., Furutani A., Tamura K., Manabe Y., Fujii N.L. (2023). Lack of Musashi-2 induces type IIa fiber-dominated muscle atrophy. FASEB J..

[49] Fujimoto Y., Tanaka S.S., Yamaguchi Y.L., Kobayashi H., Kuroki S., Tachibana M., Shinomura M., Kanai Y., Morohashi K., Kawakami K. (2013). Homeoproteins Six1 and Six4 regulate male sex determination and mouse gonadal development. Dev. Cell.

[50] Wu W., Ren Z., Li P., Yu D., Chen J., Huang R., Liu H. (2015). Six1: A critical transcription factor in tumorigenesis. Int. J. Cancer.

[51] Akiyoshi U., Takahito I., Daisuke M., Natsuko S., Tomohiro Y., Masashi S., Masahiko Y., Ryo O., Miroslav M.M., Yoko M.S. (2011). Fibrosis and adipogenesis originate from a common mesenchymal progenitor in skeletal muscle. J. Cell Sci..

[52] Riddiford N., Schlosser G. (2016). Dissecting the pre-placodal transcriptome to reveal presumptive direct targets of Six1 and Eya1 in cranial placodes. Elife.

[53] Raphaelle G., Christine L., Francois S., Soledad L., Josiane D., Jacques E.G., Kiyoshi K., Pin X.X., Robert K., Basil J.P. (2004). Six1 and Eya1 expression can reprogram adult muscle from the slow-twitch phenotype into the fast-twitch phenotype. Mol. Cell. Biol..

[54] Xiaoliang G., Yi C., Weijie W., Wenjiao L., Wenyao Z., Jiayi C., Xin F., Hui L., Di C., Daiming F. (2023). Detrimental role of Six1 in hepatic lipogenesis and fibrosis of non-alchholic fatty acid liver disease. Liver Int..

[55] Sun K., Li X., Scherer P.E. (2023). Extracellular Matrix (ECM) and Fibrosis in Adipose Tissue: Overview and Perspectives. Compr. Physiol..

[56] Wang J., Liu C., Wang T., Li S., Bai Y., Pan F., Wang J., Han J., Luo R., Wan X. (2023). Single-cell communication patterns and their intracellular information flow in synovial fibroblastic osteoarthritis and rheumatoid arthritis. Immunol. Lett..

[57] Thannickal V.J., Lee D.Y., White E.S., Cui Z., Larios J.M., Chacon R., Horowitz J.C., Day R.M., Thomas P.E. (2003). Myofibroblast differentiation by transforming growth factor-beta1 is dependent on cell adhesion and integrin signaling via focal adhesion kinase. J. Biol. Chem..

[58] Evans R.A., Tian Y.C., Steadman R., Phillips A.O. (2003). TGF-beta1-mediated fibroblast-myofibroblast terminal differentiation-the role of Smad proteins. Exp. Cell Res..

