# Supplementary material for: Six1-Eya1 Axis Governs Myofiber Remodeling and Fibrosis in Extraocular Myopathy: Insights from Single-Cell RNA Sequencing and Mesenchymal Stem Cell Therapy in Thyroid Eye Disease
Source: Cells. 2025 Oct 31;14(21):1708. doi: 10.3390/cells14211708 (PMC12608014; doi:10.3390/cells14211708)
Supplement: Supplementary file 1 [file cells-14-01708-s001.zip › cells-3911859-supplementary.pdf]

Supplementary figures

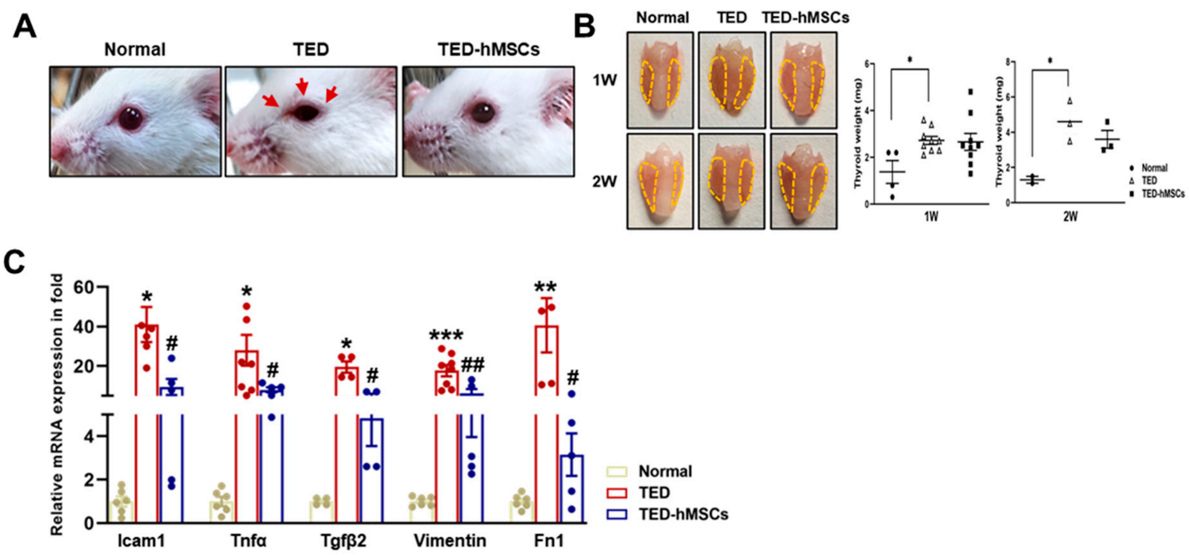

Figure S1. Features of TED in animal model. (**A**) Morphological comparison among normal, TED and TED−hMSCs groups. (**B**) Comparison of thyroid morphologies and weights between normal TED and TED−hMSCs groups at 1−2−weeks. (**C**) The relative mRNA expression of inflammation−related genes (e.g., Icam1, Tnfα, and Tgfβ2) and fibrosis−related genes (e.g., Vimentin and Fibronectin; Fn1) were analyzed by qRT−PCR in normal, TED and TED-hMSCs groups. Significantly different values between the groups are indicated with markers (*p<0.05, **p<0.01, ***p<0.001 vs normal, #p<0.05, ##p<0.01 vs TED).

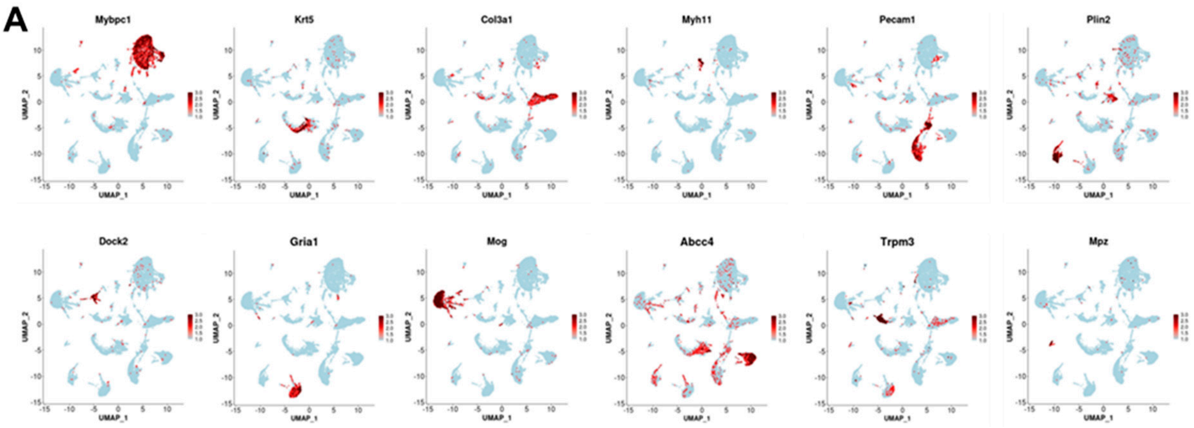

Figure S2. Single−cell RNA sequence analysis. (**A**) A feature plot showing the expression of marker genes on the UMAP representation of the map. The color scale represents z-score transformation of log2 values.

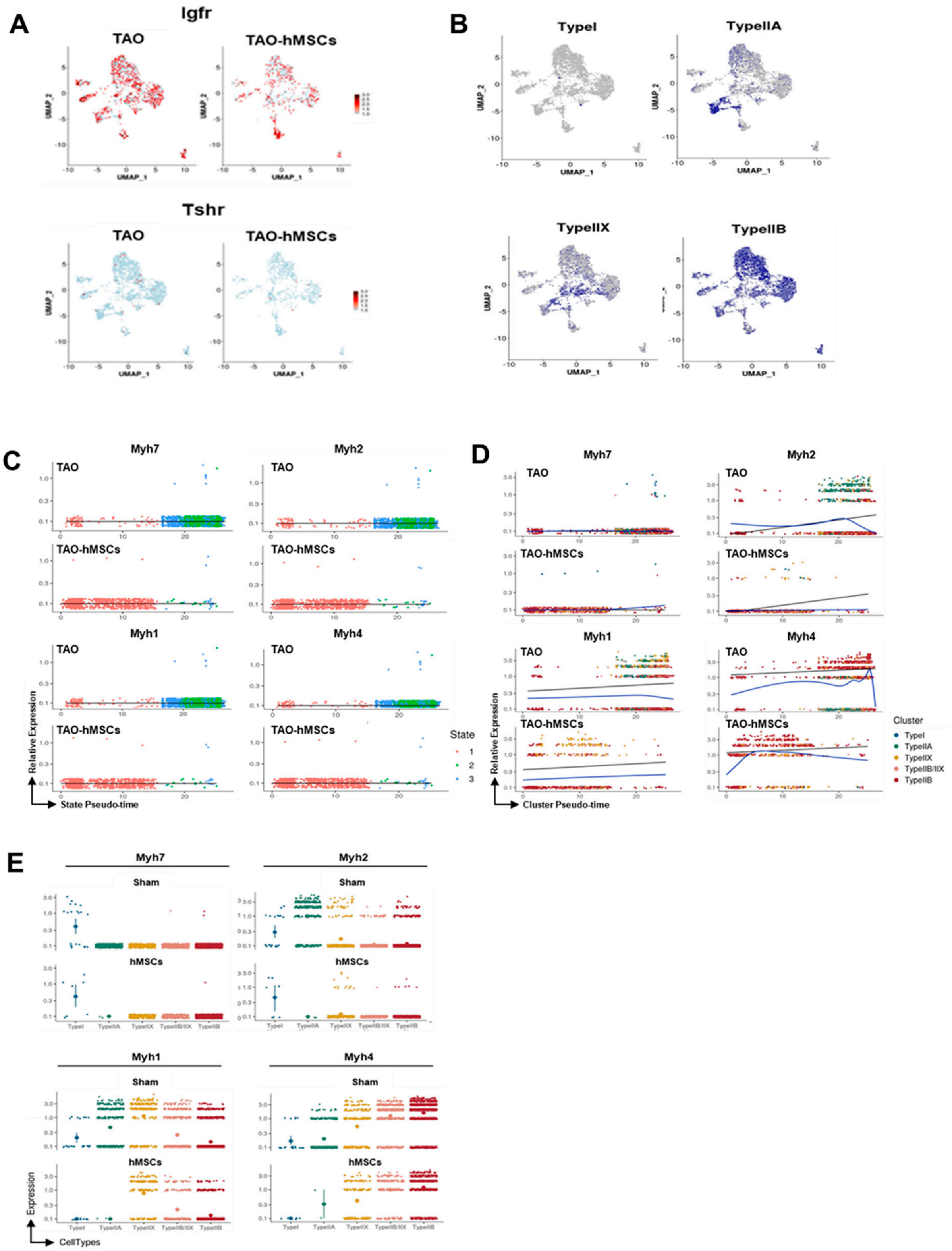

Figure S3. Single−cell RNA sequencing analysis in myocytes. (**A**) A feature plot showing the expression of TED associated marker genes in TED and TED−hMSCs. (**B**) Myocytes were divided into four subgroups using Seurat's "AddModuleScore" function. Scatter plots depict changes in the expression levels of marker genes for myofiber types over pseudotime, organized by (**C**) states and (**D**) clusters in TED and TED−hMSCs. A blue line represents

the trend in gene expression levels over pseudotime for each gene. (**E**) The expression of marker genes for myofiber types across different myocyte types is visualized using a color gradient generated by Monocle−2.

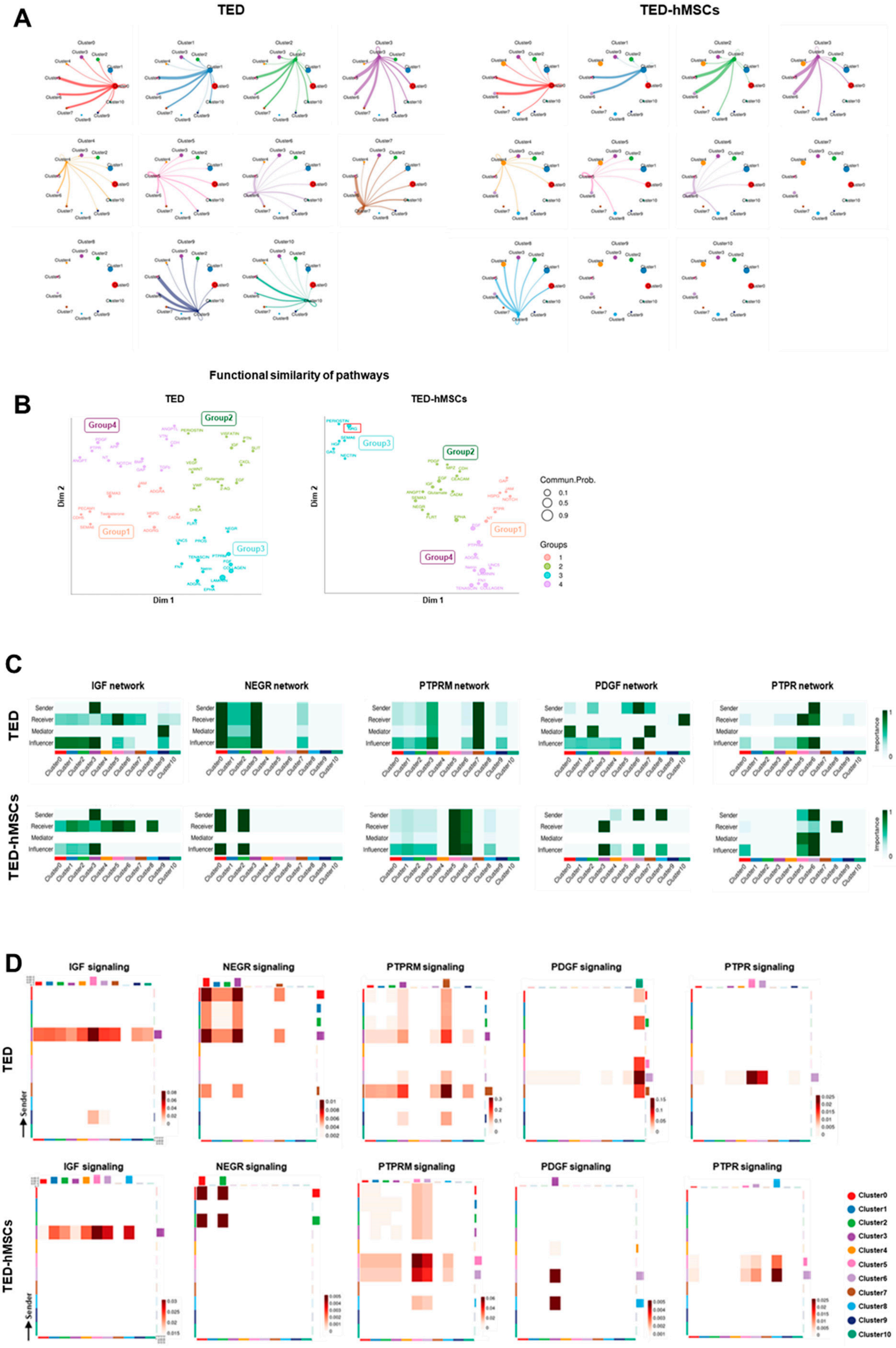
A
TED
TED-hMSCs
Functional similarity of pathways
B
TED
TED-hMSCs
Group1
Group2
Group3
Group4
Dim 1
Dim 2
Commun.Prob.
0.1
0.5
0.9
Groups
1
2
3
4
C
IGF network
NEGR network
PTPRM network
PDGF network
PTPR network
TED
TED-hMSCs
Sender
Receiver
Mediator
Influencer
Importance
D
IGF signaling
NEGR signaling
PTPRM signaling
PDGF signaling
PTPR signaling
Sender
Cluster0
Cluster1
Cluster2
Cluster3
Cluster4
Cluster5
Cluster6
Cluster7
Cluster8
Cluster9
Cluster10

Figure S4. Single−cell RNA sequencing analysis in fibroblast and myofibroblast. (**A**) Network of eleven clusters representing one−way intercellular interaction between TED and TED−hMSCs. (**B**) Signaling pathways projected onto a two-dimensional manifold according to their functional similarity. Each dot represents the communication network of one signaling pathway. Dot size is proportional to the overall communication probability. Different colors represent different groups of signaling pathways. (**C**) A heatmap showing the relative importance of each cell group based on the four network centrality measures of Figure 3A signaling network. (**D**) Heatmap showing the total interaction strength between sender and receiver clusters.

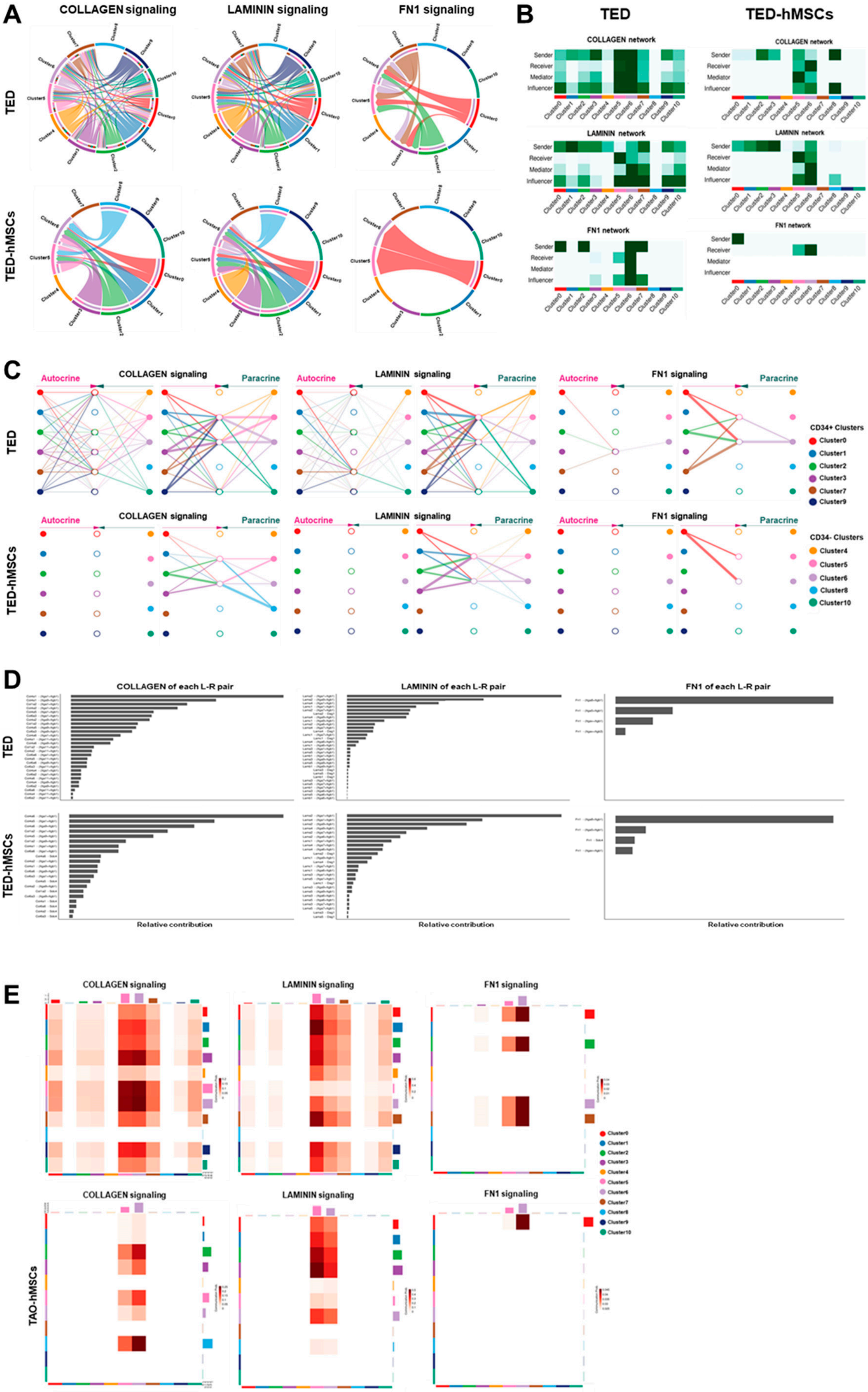

A
COLLAGEN signaling
LAMININ signaling
FN1 signaling
TED
TED-hMSCs
B
TED
TED-hMSCs
COLLAGEN network
LAMININ network
FN1 network
Sender
Receiver
Mediator
Influencer
Importance
C
Autocrine
Paracrine
COLLAGEN signaling
LAMININ signaling
FN1 signaling
TED
TED-hMSCs
CD34+ Clusters
Cluster0
Cluster1
Cluster2
Cluster3
Cluster7
Cluster9
CD34- Clusters
Cluster4
Cluster5
Cluster6
Cluster8
Cluster10
D
COLLAGEN of each L-R pair
LAMININ of each L-R pair
FN1 of each L-R pair
TED
TED-hMSCs
Relative contribution
E
COLLAGEN signaling
LAMININ signaling
FN1 signaling
TAO-hMSCs

Figure S5. ECM component signaling pathway in fibroblast and myofibroblast. (**A**) Chord diagrams representing cell-cell communication associated with ECM component signaling pathway (e.g., COLLAGEN, LAMININ and FN1). (**B**) The ECM component signaling network in fibroblast and myofibroblast in TED and TED−hMSCs. (**C**) The ECM component signaling network showing hierarchical analysis Left and right portions show the autocrine and paracrine signaling to CD34+ and CD34- clusters, respectively. Edge width represents the communication probability. (**D**) Relative contribution of each ligand−receptor pair to the overall communication network of the ECM component signaling pathway, which is the ratio of the total communication probability of the inferred network of each ligand-receptor (L−R) pair to that of the ECM components signaling pathway. (**E**) A heatmap showing the total interaction strength between sender and receiver clusters.

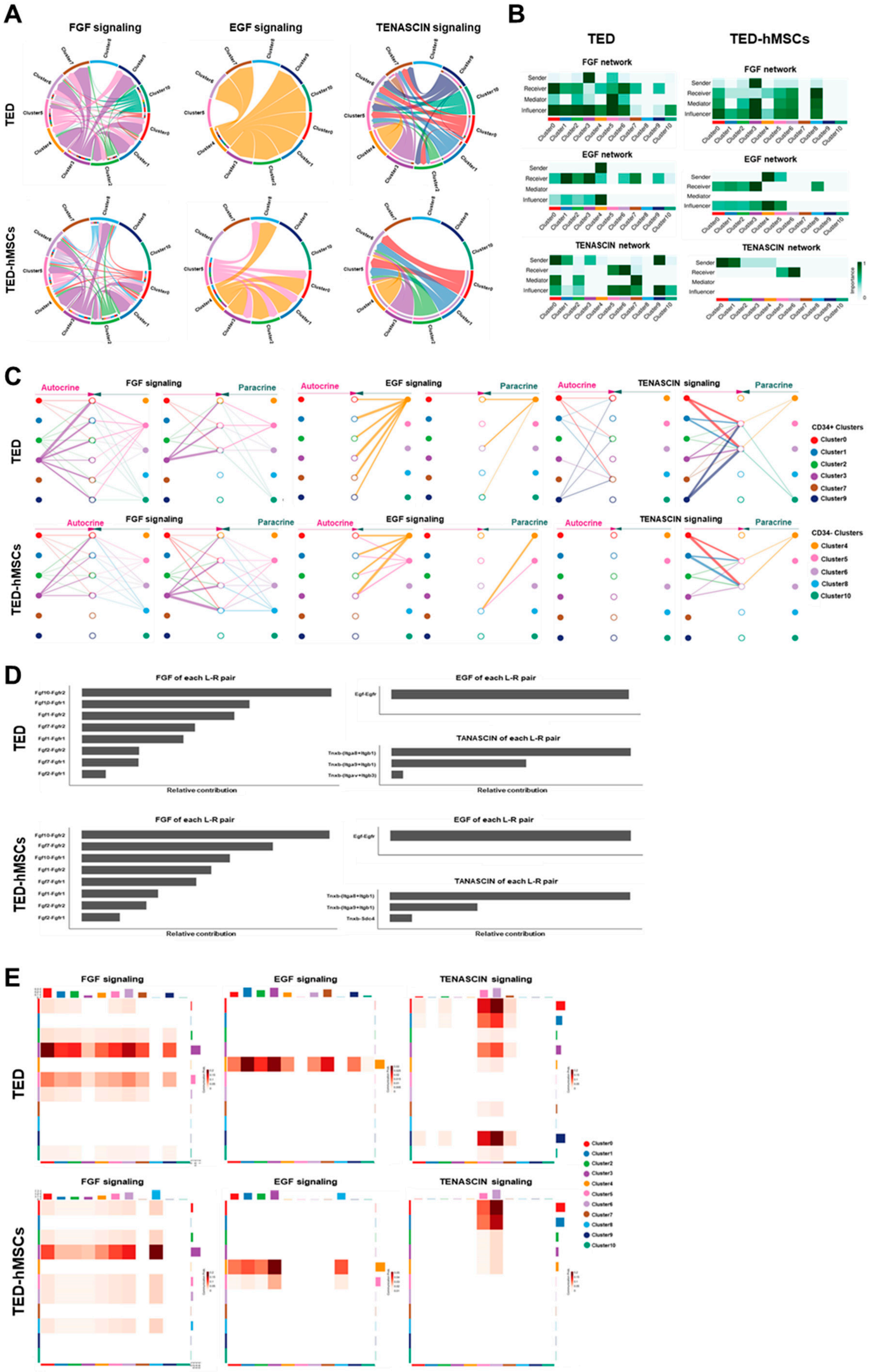
A
FGF signaling
EGF signaling
TENASCIN signaling
TED
TED-hMSCs
B
TED
TED-hMSCs
FGF network
EGF network
TENASCIN network
Sender
Receiver
Mediator
Influencer
Importance
C
Autocrine
Paracrine
FGF signaling
EGF signaling
TENASCIN signaling
CD34+ Clusters
Cluster0
Cluster1
Cluster2
Cluster3
Cluster7
Cluster9
CD34- Clusters
Cluster4
Cluster5
Cluster6
Cluster8
Cluster10
D
FGF of each L-R pair
Fgf10-Fgfr2
Fgf10-Fgfr1
Fgf1-Fgfr2
Fgf7-Fgfr2
Fgf1-Fgfr1
Fgf2-Fgfr2
Fgf7-Fgfr1
Fgf2-Fgfr1
Relative contribution
EGF of each L-R pair
Egf-Egfr
TANASCIN of each L-R pair
Tnxb-(Itga8+Itgb1)
Tnxb-(Itga9+Itgb1)
Tnxb-(Itgav+Itgb3)
Relative contribution
FGF of each L-R pair
Fgf10-Fgfr2
Fgf7-Fgfr2
Fgf10-Fgfr1
Fgf1-Fgfr2
Fgf7-Fgfr1
Fgf1-Fgfr1
Fgf2-Fgfr2
Fgf2-Fgfr1
Relative contribution
EGF of each L-R pair
Egf-Egfr
TANASCIN of each L-R pair
Tnxb-(Itga8+Itgb1)
Tnxb-(Itga9+Itgb1)
Tnxb-Sdc4
Relative contribution
E
FGF signaling
EGF signaling
TENASCIN signaling
Cluster0
Cluster1
Cluster2
Cluster3
Cluster4
Cluster5
Cluster6
Cluster7
Cluster8
Cluster9
Cluster10

Figure S6. Inflammatory signaling pathway in fibroblast and myofibroblast. (**A**) Chord diagrams representing cell−cell communication associated with inflammation signaling pathway (e.g., FGF, EGF and TENASCIN). (**B**) The inflammation signaling network showing hierarchical analysis. (**C**) Ligand−Receptor pairs and (**D**) Relative contribution of each ligand−receptor pair to the overall communication network of the inflammation signaling pathway. (**E**) A heatmap showing the total interaction strength between sender and receiver clusters.

Table S1. Basic characteristics of patients

No	Gender	Age	Type	Site
#C1	M	20	Normal	Rectus muscle
#C2	M	54	Normal	Rectus muscle
#C3	F	66	Normal	Rectus muscle
#T1	F	32	TED	Levator muscle
#T2	F	36	TED	Levator muscle
#T3	M	31	TED	Muller muscle

Table S2. Human primer sequences using qRT−PCR

Genes	Primer Sequence
IGF-1R	Forward 5’- AGAAGGAGGAGGCTGAATAC -3’<br>Reverse 5’- GGTCGGTGATGTTGTAGGT -3’
TSHR	Forward 5’- GAGTTTCCTTCACCTCACACGG -3’<br>Reverse 5’- CTGCTCTCATTACACATCAAGGAC -3’
DESMIN	Forward 5’- TCCAGTCCTACACCTGCGAGAT -3’<br>Reverse 5’- CGCAATGTTGTCCTGGTAGCCA -3’
TGFβ1	Forward 5’- CTGGACACCAACTATTGC -3’<br>Reverse 5’- CTTCCACCCGAGGTCCTT -3’
TGFβ2	Forward 5’- ATTGCCCTCCTACAGACTTGAG -3’<br>Reverse 5’- CAGCACAGAAGTTGGCCATTGTA -3’
α-SMA	Forward 5’- CTCCCAGGGCTGTTTTCCCA -3’<br>Reverse 5’- CCATGTCGTCCCAGTTGGTG -3’
FIBRONECTIN	Forward 5’- CCAAGAAGGGCTCGTGTG -3’<br>Reverse 5’- TGGCTGGAACGGCATCA -3’
HAS1	Forward 5’-CTGCGATACTGGGTAGCCTTCA-3’<br>Reverse 5’-CCAGGAACTTCTGGTTGTACCAG-3’
HAS2	Forward 5’- GTCATGTACACAGCCTTCAGAGC -3’<br>Reverse 5’- ACAGATGAGGCTGGGTCAAGCA -3’
HAS3	Forward 5’- AGCACCTTCTCGTGCATCATGC -3’<br>Reverse 5’- TCCTCCAGGACTCGAAGCATCT -3’
TSG6	Forward 5’- TCACCTACGCAGAAGCTAAGGC -3’<br>Reverse 5’- TCCAACTCTGCCCTTAGCCATC -3’
SIX1	Forward 5’- TTACGCAGGAGCAAGTGGCG -3’<br>Reverse 5’- CGCTCTCGTTCTTGTGCAGG -3’
EYA1	Forward 5’- TGGCATCACCAGCCAAGCAGTT -3’<br>Reverse 5’- CCATCTGAACCTCGACGCAATC -3’
SOX6	Forward 5’- TAAGCAACTGATGAGGTCTC -3’<br>Reverse 5’- AGGCGATGGTGTGGTAGTT -3’
NFIX	Forward 5’- GAGAGCCCTGTTGATGACG -3’<br>Reverse 5’- CTGCAGAAGTCCAGCTTTCC -3’
MYH7	Forward 5’- GGAGTTCACACGCCTCAAAGAG -3’<br>Reverse 5’- TCCTCAGCATCTGCCAGGTTGT -3’
MYH2	Forward 5’- GGAGGACAAAGTCAACACCCTG -3’<br>Reverse 5’- GCCCTTTCTAGGTCCATGCGAA -3’
MYH1	Forward 5’- GCTGGCTAAGACCGAGGCAAAA -3’<br>Reverse 5’- CCTTTCCTCTGCATCAGCCAAG -3’
MYH4	Forward 5’- GACAGCCAAGAAGAGGAAACTGG -3’<br>Reverse 5’- ACCTGCCATCTCTTCTGTGAGG -3’
PPARγ	Forward 5’- TTGACCCAGAAAGCGATTCC -3’<br>Reverse 5’- AAAGTTGGTGGGCCAGAATG -3’
C/EBPα	Forward 5’- TGTATACCCCTGGTGGGAGA -3’<br>Reverse 5’- TCATAACTCCGGTCCCTCTG -3’
LEPTIN	Forward 5’- GGTTGCAAGGCCCAAGAA -3’<br>Reverse 5’- ACATAGAAAAGATAGGGCCAGC -3’

MYH3	Forward 5’- CTGGAGGATGAATGCTCAGAGC -3’<br>Reverse 5’- CCCAGAGAGTTCCTCAGTAAGG -3’
IGF	Forward 5’- CATGTCCTCCTCGCATCTCT -3’<br>Reverse 5’- GGTGCGCAATACATCTCGAG -3’
NRG2	Forward 5’- TCCCCCTCCTCTGATGATTAAA -3’<br>Reverse 5’- CAGGAAGCCTTCTTGCCTCATA -3’
FGFR1	Forward 5’- GCAACGTGGAGTTCATGTGTAAG -3’<br>Reverse 5’- TTGTCTGGGCCAATCTTGCT -3’
MMP2	Forward 5’- TCAAGGGCATTCAGGAGCTCTA -3’<br>Reverse 5’- CTGTTTGCAGATCTCAGGAGTGA -3’
COLLAGEN I	Forward 5’- TCCTGCCGATGTCGCTATC -3’<br>Reverse 5’- CAAGTTCCGGTGTGACTCGTG -3’
COLLAGEN III	Forward 5’- TTTCCCAGGTCAAGATGGTC -3’<br>Reverse 5’- CTTCAGCACCTGTCTCACCA -3’
18S rRNA	Forward 5’- GAAACTGCGAATGGCTCATTAAATCA -3’<br>Reverse 5’- CCCGTCGGCATGTATTAGCTCT -3’

Table S3. Mouse primer sequences using qRT−PCR

**Genes**	**Primer Sequence**
Icam1	Forward 5’- AGCATTTACCCTCAGCCACT -3’<br>Reverse 5’- TGAAGTCAGCGTTTCTTGGC -3’
Tnfα	Forward 5’- ACCCTCACACTCACAAACCA -3’<br>Reverse 5’- GGCAGAGAGGAGGTTGACTT -3’
Tgfβ2	Forward 5’- AGAAGGATCGGCAGAGGTCT -3’<br>Reverse 5’- AGCTCGGTCCTTCAGATCCT -3’
Vimentin	Forward 5’- CGCTTTGCCAACTACATCGA -3’<br>Reverse 5’- CCTCCTGCAATTTCTCTCGC -3’
Fibronectin	Forward 5’- TCCCGGGCAGAAAGTACATT -3’<br>Reverse 5’- TTCAGGGAGGTTGAGCTCTG -3’
